# Supplementary material for: A Guide for the Design of Evolve and Resequencing Studies
Source: Mol Biol Evol. 2013 Nov 9;31(2):474–83. doi: 10.1093/molbev/mst221 (PMC3907048; doi:10.1093/molbev/mst221)
Supplement: Supplementary Data [file supp_31_2_474__index.html]

A Guide for the Design of Evolve and Resequencing Studies — A Guide for the Design of Evolve and Resequencing Studies — Supplementary Data 

# A Guide for the Design of Evolve and Resequencing Studies

## Supplementary Data

files

**Files in this Data Supplement:**

- Supplementary Data - pdf file
- Supplementary Data - xlsx file
