# Supplementary material for: A Guide for the Design of Evolve and Resequencing Studies
Source: Mol Biol Evol. 2013 Nov 9;31(2):474–83. doi: 10.1093/molbev/mst221 (PMC3907048; doi:10.1093/molbev/mst221)
Supplement: Supplementary Data [file supp_mst221_supplement.pdf]

Supplement to a guide for the design of evolve and resequencing studies

Robert Kofler[1], and Christian Schlötterer*[1]

[1]Institut für Populationsgenetik, Vetmeduni Vienna, Veterinärplatz 1, 1210 Wien, Austria

Email: Robert Kofler - rokofler@gmail.com; Christian Schlötterer*- christian.schloetterer@vetmeduni.ac.at;

*Corresponding author

Contents

1 Supplementary Results **2**

1.1 Base population 2

1.2 Test statistic 5

1.3 Low recombining regions 6

1.4 Number of selected loci 8

1.5 Number of replicates 10

1.6 Number of haploid genomes in the base population 12

1.7 Tradeoff between population size and replicate numbers 14

1.8 Influence of the test statistic 16

1.9 Influence of the number of selected loci 17

1.10 Influence of the dominance coefficient (h) 18

1.11 Influence of the starting allele frequency 19

1.12 Influence of mixing beneficial loci with different selection coefficient 21

1.13 Influence of estimating the allele frequency with Pool-Seq 23

2 Supplementary figures **25**

3 Supplementary Material and Methods 39
3.1 Estimating the efficacy of selection 39
3.2 Simulating the sampling properties of Pool-Seq 39

1 Supplementary Results

1.1 Base population

As a base population for the simulation of experimental evolution we simulated 8,000 haploid genomes with fastsimcoal v1.1.8 [1] that reproduce the pattern of natural variation of a *D. melanogaster* population captured in Vienna in fall 2010 [2]. The average number of SNPs and the nucleotide diversity (Tajima's π) for this simulated base population in windows of 10,000 bp can be found in Figure 1. As we excluded the X-chromosome from the analysis we only show results for the chromosomes 2L, 2R, 3L, and 3R; Only a subset of the 8,000 haploid genomes was used for most of the simulations, we therefore show the number of SNPs in the base population dependent on the number of sampled haploid genomes in table 1. Figure 2 shows the allele frequency spectrum for the ancestral and the derived allele in the simulated base population excluding the low recombining regions (<1 cM/Mb). For the simulations beneficial loci were randomly chosen, where either the ancestral or the derived allele was randomly picked as the beneficial one. To increase the probability of detecting the selected loci, the starting frequency of selected alleles was not allowed to exceed 80% (excluded alleles are shaded in gray). Figure 2 also demonstrates that the majority of the beneficial alleles will be derived alleles.

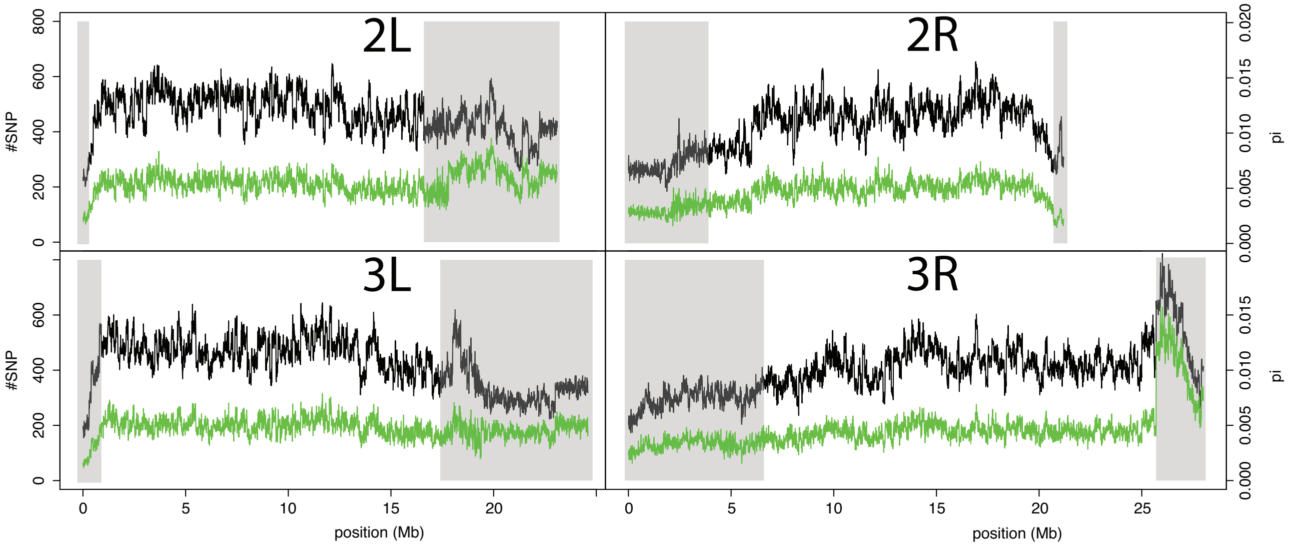

Figure 1: Density of SNPs (black) and Tajima's π (green) in windows of 10.000bp for the simulated base population of *D.melanogaster*; Shaded grey areas represents regions with a low recombination rate (<1cM/Mb)

Table 1: Average number of SNPs in the base population dependent on the number of sampled haploid genomes (h.gen.), including (incl. l.r) and excluding low recombining regions (excl. l.r)

h.gen.	incl. l.r.	excl.l.r
8,000	4,126,838	3,129,057
4,000	3,836,061	2,907,179
2,000	3,545,522	2,685,047
1,000	3,252,873	2,461,301
500	2,960,875	2,236,738
200	2,574,225	1,939,075
100	2,281,870	1,711,996

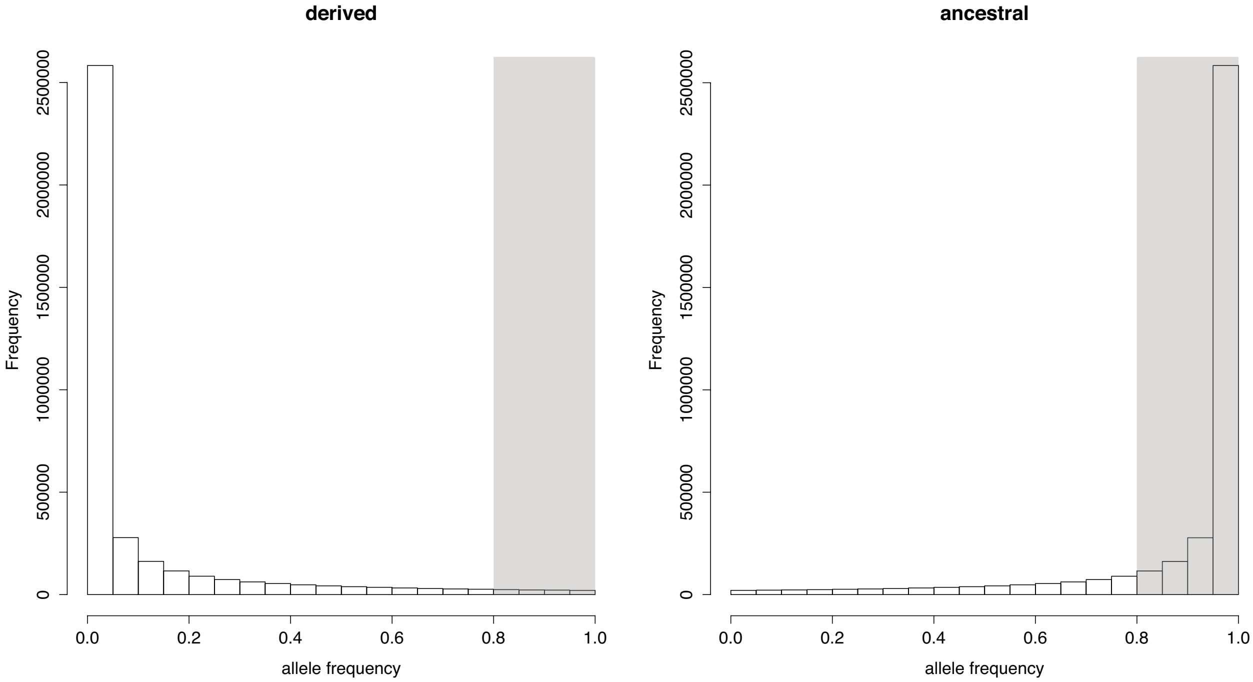

Figure 2: Allele frequency spectrum for the ancestral and the derived allele in the base population with 8,000 haploid genomes excluding the low recombining regions (<1 cM/Mb). Shaded gray areas indicate alleles that are not available as beneficial ones as beneficial alleles were not allowed to exceed a frequency of 0.8

1.2 Test statistic

To evaluate the performance of different evolve and resequencing (E&R) designs a suitable test-statistic for the identification of selected loci is required. Recently several test statistics have been suggested such as the Cochran-Mantel-Haensel test (CMH-test) [3], diffStat [4], association statistic [5] and the F_{ST} [6].

We evaluated these test statistics with forward simulations resembling typical E&R studies. We used a population of 1,000 individuals and randomly picked 100 beneficial loci ($s = 0.1$; $h = 0.5$) to perform forward simulations for 60 generations with 3 biological replicates. Confidence estimates are obtained by repeating this protocol 10 times. The test statistics performed significantly differently [supplementary fig. 3; Kruskal-Wallis rank sum test with AUC (area under the ROC curve); $\chi^2 = 22.83$; $p = 4.3e - 05$]. The CMH-test showed the highest power to identify beneficial loci ($AUC = 0.8011$; $SE = 0.0069$) followed by the average pairwise F_{ST} ($AUC = 0.7759$; $SE = 0.0063$), the association statistic ($AUC = 0.7735$; $SE = 0.0074$) and the diffStat ($AUC = 0.7254$; $SE = 0.0057$). For this reason we used the CMH-test to identify beneficial loci in this study.

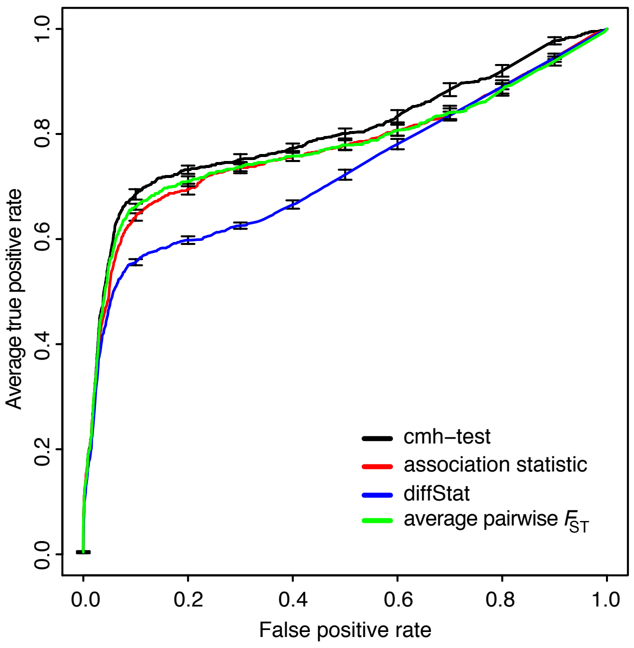

Figure 3: Comparision of the test statistics

1.3 Low recombining regions

High levels of linkage disequilibrium in low recombining regions may prevent the identification of beneficial loci. Excluding low recombining regions from the analysis, therefore, may improve the power to identify selected loci with the tradeoff that beneficial loci lying within low recombining regions cannot be detected.

To test this strategy we used the results of the simulations mentioned above (100 selected SNPs; $s = 0.1$; $h = 0.5$; $N = 1000$; homozygous individuals) and generated two data sets: one including low recombining regions ($< 1cM/Mbp$) and one excluding low-recombining regions. As E&R studies aim to identify a set of beneficial loci containing a minimum of false positives we evaluated the performance at a low false positive rate ($FPR < 0.01$) with the partial area under the curve ($pAUC$; see main text). At a low FPR the data set excluding the low recombining region had a significantly better performance than the data set that included the low recombining regions (Fig. 4 B; Wilcoxon rank sum test with pAUC; $W = 93$; $p = 0.00048$).

When evaluating the performance of the whole ROC curve, the data set that included the low recombining regions had the best performance (Figure 4 A; Wilcoxon rank sum test with AUC; $W = 100$, $p = 1.1e - 05$). This is not surprising as beneficial loci lying within low recombining regions cannot be identified with the data set that excluded the low recombining regions. As performance at a low FPR is most relevant for E&R studies we recommend excluding low recombination regions from the analyses and followed this strategy also in our study.

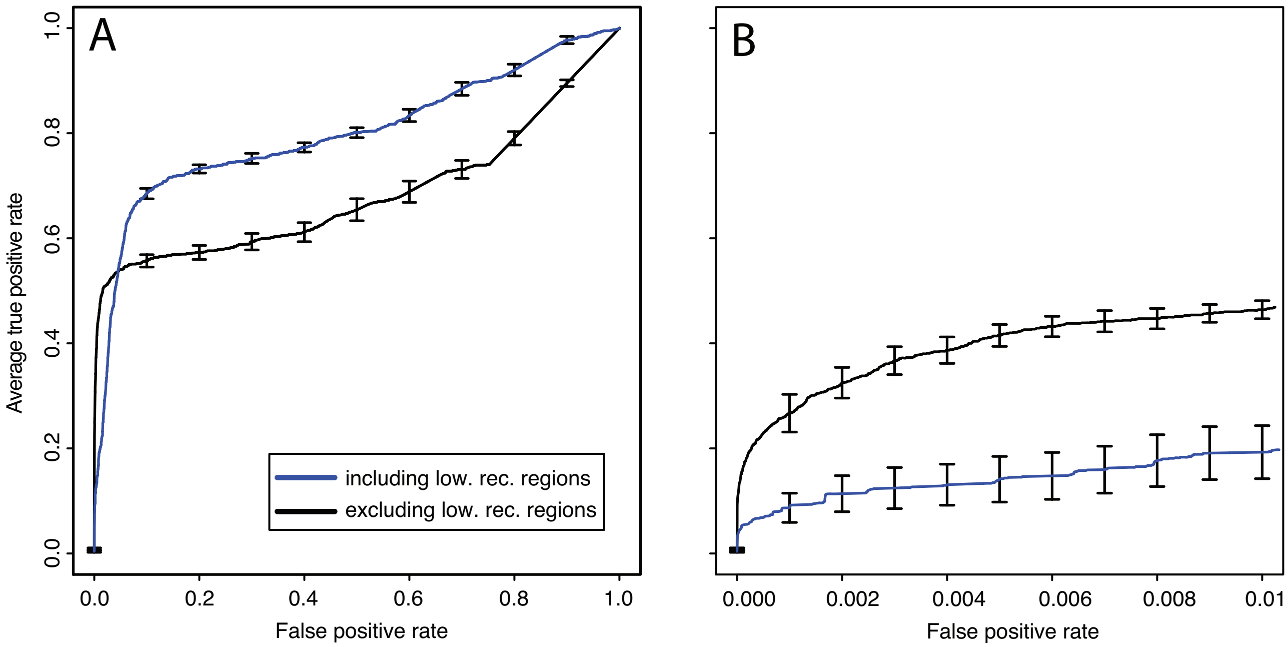

Figure 4: Influence of low recombining ($< 1cM/Mbp$) regions at a FPR of 1.0 (A) and 0.01 (B)

1.4 Number of selected loci

Given that forward simulations are highly CPU intensive, in particular for large population sizes, we were interested in minimizing the number of simulations. To obtain a sufficient number of observations with a moderate number of simulation runs, we opted for simulating multiple selected loci. One possible complication of this strategy is that too many targets of selection will interfere with each other, precluding generalization of the results. We explored how the number of selected loci influences the efficacy of selection by simulations (see supplementary material and methods). We measured the efficacy of selection first by a reduction in the probability of fixation (p), and second by a reduction in the effective population size (N_e) in the first generation, due to variance of offspring [7]. The efficacy of selection decreased with increasing numbers of selected loci for both strongly ($s = 0.1$) and weakly ($s = 0.025$) selected ones (fig. 5). The choice of an optimal number of selected loci is somewhat arbitrary. Based on supplementary fig. 5 we used 150 beneficial loci for our study. We argue that this is the largest number of beneficial loci that can be simultaneously selected without disproportionally reducing the efficacy of selection. From these simulations we also estimated the probability of fixation for 150 beneficial loci as $p = 0.673$ ($s = 0.1$) and $p = 0.633$ ($s = 0.025$).

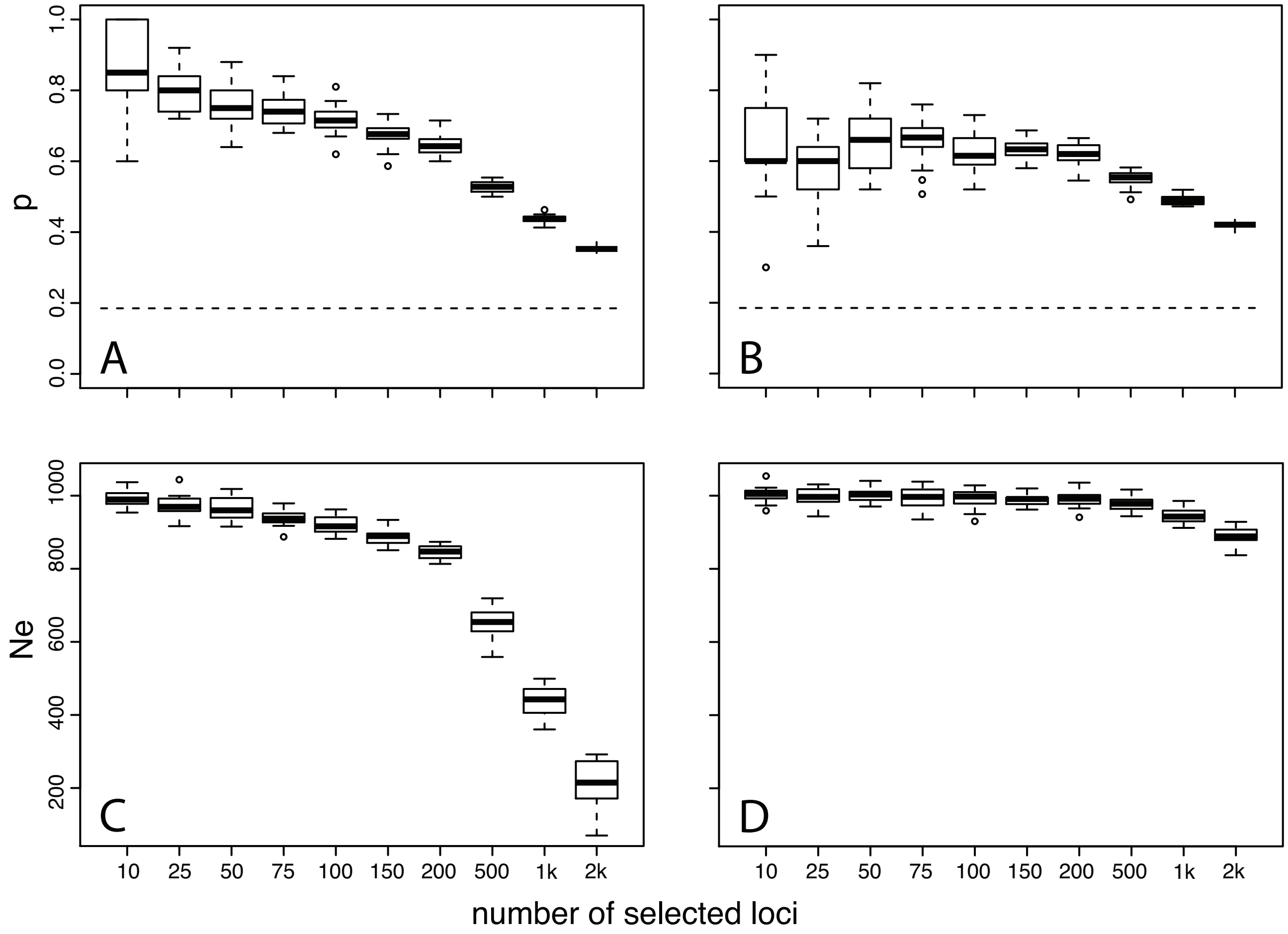

Figure 5: Efficacy of selection as estimated by the probability of fixation (p) and the effective population size (N_e) for different numbers of selected loci; Twenty replicate simulations have been performed for each number of selected loci; A: p for $s = 0.1$; B: p for $s = 0.025$; C: N_e for $s = 0.1$; D: N_e for $s = 0.025$; The dashed lines indicates the expected probability of fixation under neutrality

1.5 Number of replicates

We found that for a sufficient number of replicates weakly selected loci are more easily identified than strongly selected loci. To shed more light on this observation, we used the signal-to-noise ratio (SNR). The signal is defined as the average log-transformed p-value of selected loci and the noise as the average log-transformed p-value of not selected loci [signal: $SIG = 1/n_s * \sum_{n_s} -log_{10}(p_{ns})$ where n_s are the selected loci and p the p-value of the CMH-test; noise: $NOI = 1/n_n * \sum_{n_n} -log_{10}(p_{nn})$ where n_n are the non-selected loci and p the p-value of the CMH-test; signal-to-noise ratio (SNR): $SNR = SIG/NOI$]. We found that the SNR of weakly selected loci increases more with additional replicates than the SNR of strongly selected loci (supplementary fig. 6 A). When investigating the signal and the noise separately (supplementary fig. 6 B) we observed that the signal shows a similar slope for both selection coefficients whereas the noise is increasing more slowly for the weakly selected loci thus causing the steeper slope of the SNR. We interpret this observation to be the result of neutral variants linked to weakly selected loci having more time to recombine onto not-selected haplotypes relative to variants linked to strongly selected haplotypes, which will fix rapidly (or at least rise to high frequencies). Note that the noise increases with the number of replicates as consistent changes in allele frequencies over increasing number of replicates - as for example caused by hitchhiking alleles - will yield higher values of the CMH-test. This is also confirmed by the number of fixed loci in the evolved populations, where we found a higher number of fixed loci for strongly selected loci ($s = 0.1$: $\overline{x} = 1202882.0$, $SE = 2106.9$; $s = 0.025$: $\overline{x} = 927699.4$, $SE = 503.4$; Wilcoxon rank sum test: $W = 40000$; $p < 2.2e - 16$).

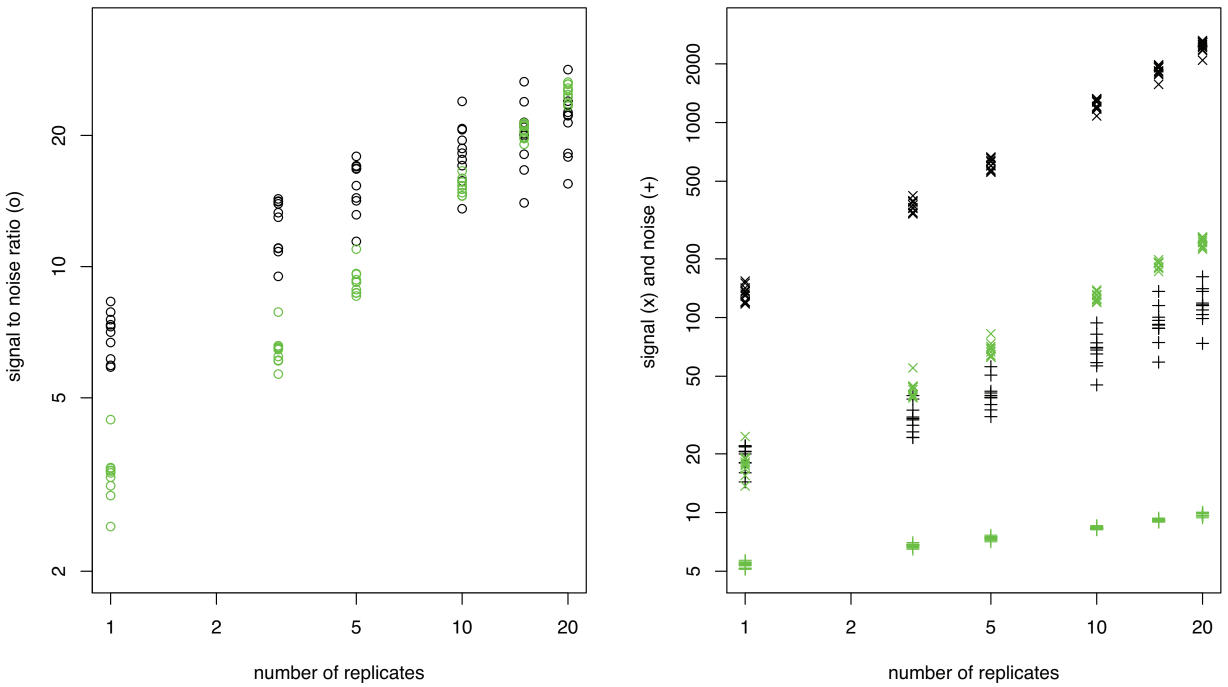

Figure 6: Signal-to-noise ratio for strongly (black; $s = 0.1$) and for weakly selected loci (green; $s = 0.025$) dependent on the number of replicates. A: signal-to-noise ratio; B: signal and noise separately; 'o': signal-to-noise ratio; 'x': signal; '+': noise

1.6 Number of haploid genomes in the base population

Our simulations showed that the number of haploid genomes in the base population influenced the power to identify beneficial loci more for strongly selected loci than for weakly selected ones. We again used the SNR to investigate this observation (supplementary fig. 21). For strongly selected loci the SNR increases constantly with the number of chromsomes, whereas for weakly selected loci the increase of the SNR levels off at about 200 haploid genomes (supplementary fig. 7 A). A similar behavior was seen with the ROC curve (supplementary fig. 20; or see main text). The signal decreases for both selection coefficients at a similar rate (supplementary fig. 7 B). This behavior can be explained by how we assigned selected loci. Since selected loci are randomly chosen, most of them are singletons in the chromosomes used to establish the base population. With a population size of 1,000 individuals, the number of haploid genomes affects the starting frequency, e.g. with 100 haploid genomes the lowest possible starting frequency is 1%, but with 1,000 haploid genomes it is 0.1%. This results in less pronounced allele frequency differences between base and evolved populations and therefore in a lower signal.

As a lower limit, the noise can not be reduced beyond the background level caused by neutral genetic drift (red crosses in supplementary fig. 7 B). For strongly selected loci the noise starts at a high level (due to linkage to a strong signal) and drops constantly with increasing chromosome numbers but does not approach the lower limit imposed by genetic drift. In contrast the noise of weakly selected loci starts at a low level and decreases slowly with increasing chromosome numbers and eventually reaches background noise levels at approximately 200 chromosomes (supplementary fig. 7). As no further reduction of the noise beyond background levels is possible the increase of the SNR of weakly selected loci stops at approximately 200 chromosomes. This observation is most likely explained by neutral variants linked to strongly selected loci having less opportunity to recombine onto neutral haplotypes before becoming fixed (or at least reaching high frequencies) than variants linked to weakly selected loci. Hence, the identification of strongly selected loci benefits more from unlinking selected loci and neutral loci in the base population.

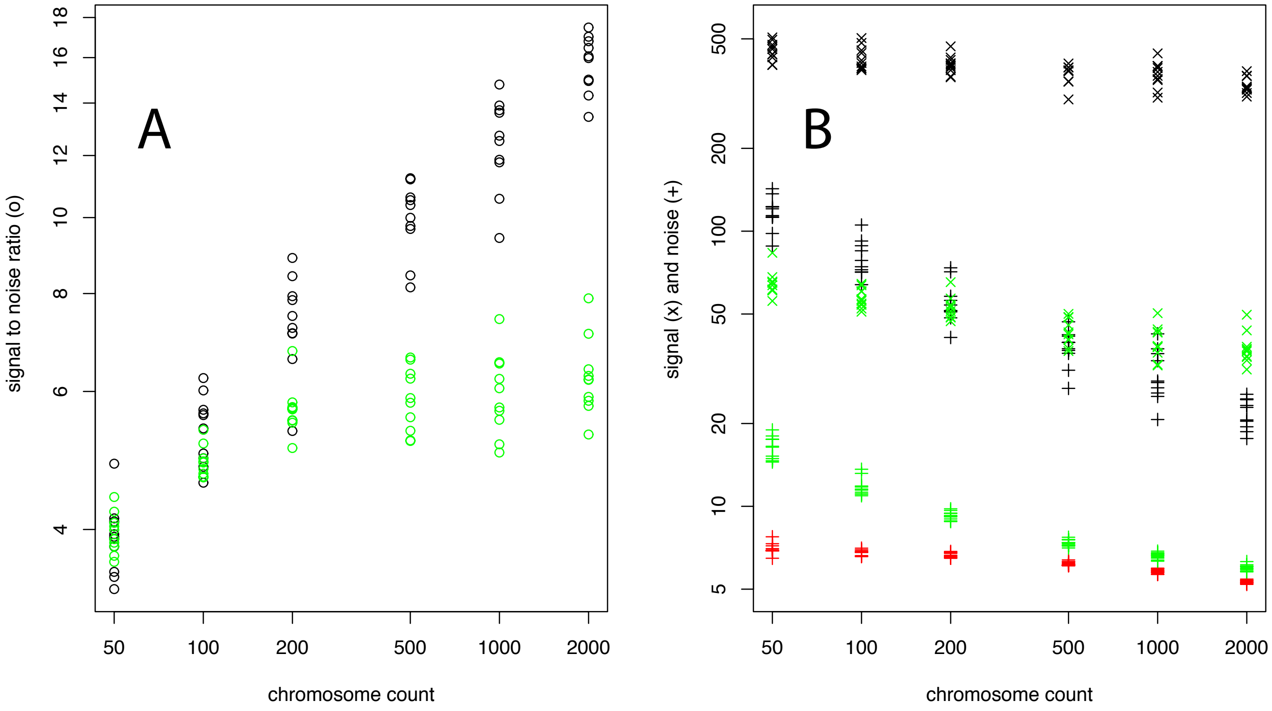

Figure 7: Signal-to-noise ratio for strongly (black; $s = 0.1$) and for weakly selected loci (green; $s = 0.025$) dependent on the number of haploid genomes in the base population. A: signal-to-noise ratio; B: signal and noise separately; 'o': signal-to-noise ratio; 'x': signal; '+': noise; red: noise from neutral simulations

1.7 Tradeoff between population size and replicate numbers

Our analyses showed that varying the population size at the expense of the number of replicates had a particularly strong effect for the identification of strongly selected loci. We analyzed this result further using the SNR. For strongly selected loci the SNR decreases with the population size, whereas for weakly selected ones the SNR does not change much (supplementary fig. 8 A). A similar behavior was seen for the ROC curves (supplementary fig. 22; supplementary fig. 23). For strongly selected loci the signal is constant whereas the noise decreases with decreasing population sizes (supplementary fig 8 B). Hence, we conclude that decreasing levels of noise are responsible for the increasing power to identify strongly selected loci when using more replicates at the cost of a decreasing population size. One explanation could be that the reduction in $N_e * s$ caused by the lower population size will increase the influence of genetic drift. Due to the stochastic nature of drift, this will retard the rapid changes in allele frequencies and thus offer more opportunities for variants linked to strongly selected sites to recombine onto neutral haplotypes.

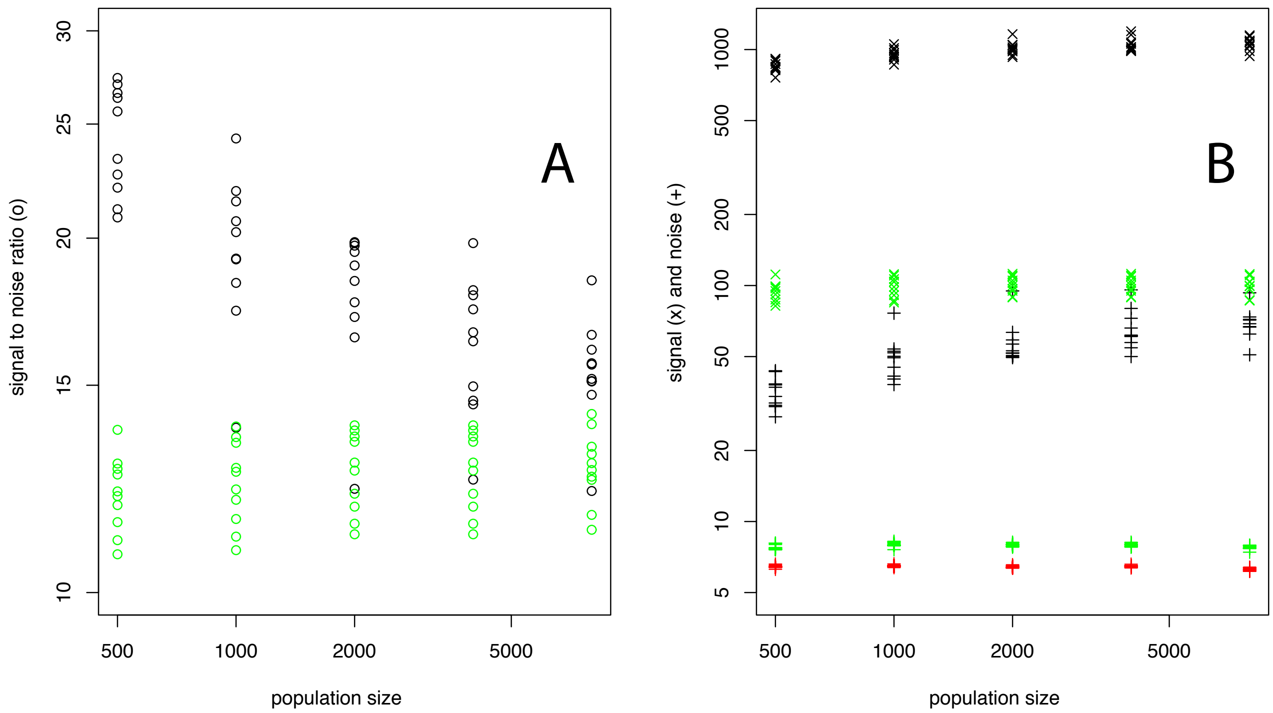

Figure 8: Signal-to-noise ratio for strongly (black; $s = 0.1$) and for weakly selected loci (green; $s = 0.025$) dependent on the combination of population size and number of replicates. A: signal-to-noise ratio ; B: signal and noise separately; 'o': signal-to-noise ratio; 'x': signal; '+': noise; red: noise from neutral simulations

1.8 Influence of the test statistic

We used the CMH-test for the identification of selected loci as this test had the best performance (supplementary results 1.2). To test the robustness of our conclusions we reanalyzed the influence of the number of replicates on weakly selected loci with the two test statistics having the best performance after the CMH-test - the association statistic and the average pairwise F_{ST}. (supplementary fig. 3) - and only found minor differences (supplementary fig. 9; for the CMH-test results see main text fig. 2 B). These results are not surprising as all of the evaluated test statistics operate on the same principle: the quantification of allele frequency differences between the base and the evolved populations.

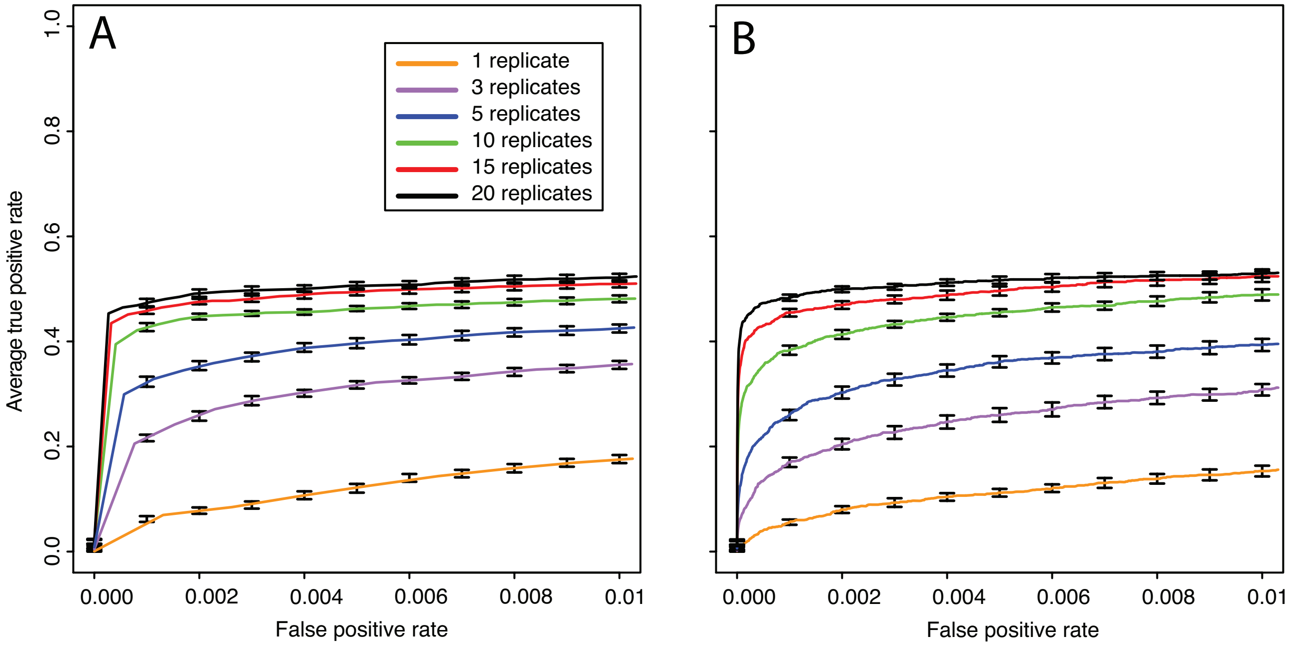

Figure 9: Influence of the test statistic on the results. The influence of the number of replicates has been reexamined for weakly selected loci ($s = 0.025$) with the association statistic (A) and the average pairwise F_{ST} (B)

1.9 Influence of the number of selected loci

We used 150 beneficial loci in our simulations, as we found this to be the largest number of loci that can be included in the simulations without disproportionally reducing the efficacy of selection (supplementary results 1.4). To further test the influence of the number of selected loci we performed simulations with varying numbers of beneficial loci ($10 - 2000$) using default conditions. We found that the number of beneficial loci has a significant influence on the power to identify beneficial loci ((Kruskal-Wallis rank sum test with pAUC; $s = 0.1$: $\chi^2 = 93.19$, $p = 3.7e - 16$; $s = 0.025$: $\chi^2 = 67.37$, $p = 5.0e - 11$), where the effect is most pronounced for strongly selected loci (Fig. 10). This is in good agreement with the simulations estimating the influence of the number of beneficial loci on the efficacy of selection (supplementary results 1.4), where we also found a more pronounced effect for strongly selected loci. However, these results suggest that the power to identify strongly selected loci in an E&R studies, where only a few (< 150) loci are strongly selected, may be moderately higher as shown in our work.

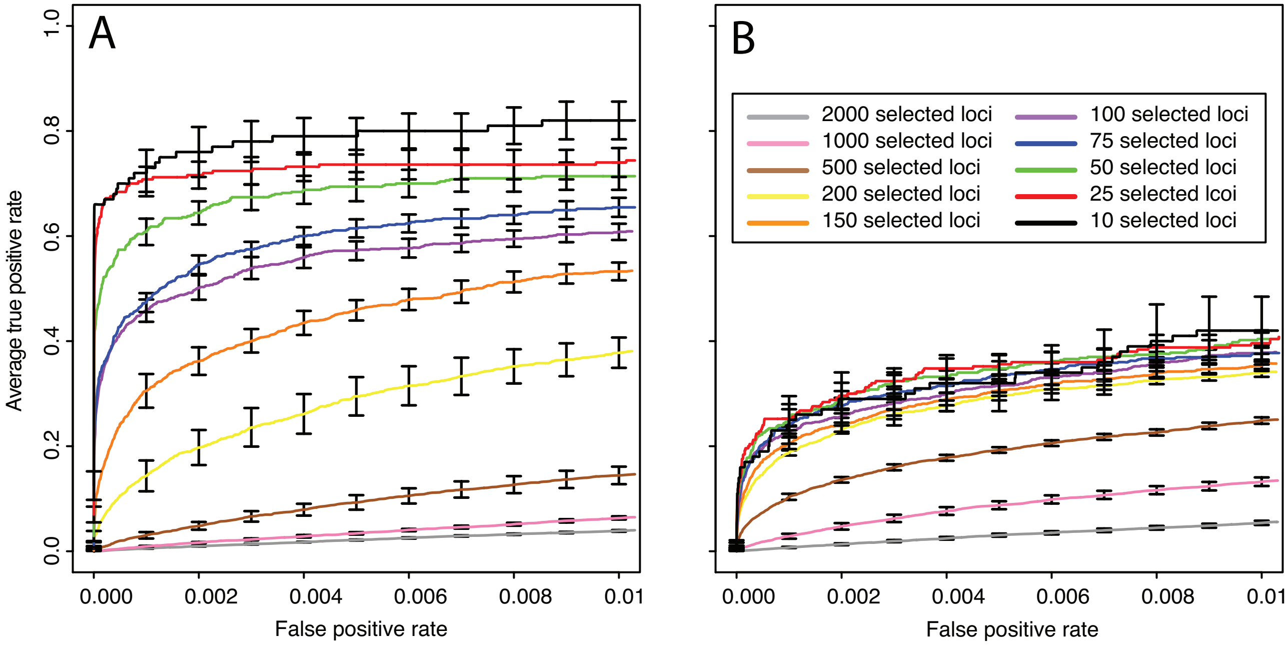

Figure 10: Influence of the number of selected loci on identification of beneficial loci with selection coefficients $s = 0.1$ (A) and $s = 0.025$ (B)

1.10 Influence of the dominance coefficient (h)

To evaluate the influence of the dominance coefficient (h) on the power to identify beneficial loci we used default conditions and varied the dominance coefficient from $h = 0.0$ to $h = 1.0$. We found that large dominance coefficients ($1.0 \geq h > 0.5$: dominant) result in an increased power to identify beneficial loci whereas low dominance coefficients ($h < 0.5$: recessive) result in a decreased performance (supplementary fig. 11). The influence of the dominance coefficient is, however, only significant for weakly selected loci (Kruskal-Wallis rank sum test with pAUC; $s = 0.1$: $\chi^2 = 7.03$, $p = 0.13$; $s = 0.025$: $\chi^2 = 35.84$, $p = 3.1e-07$).

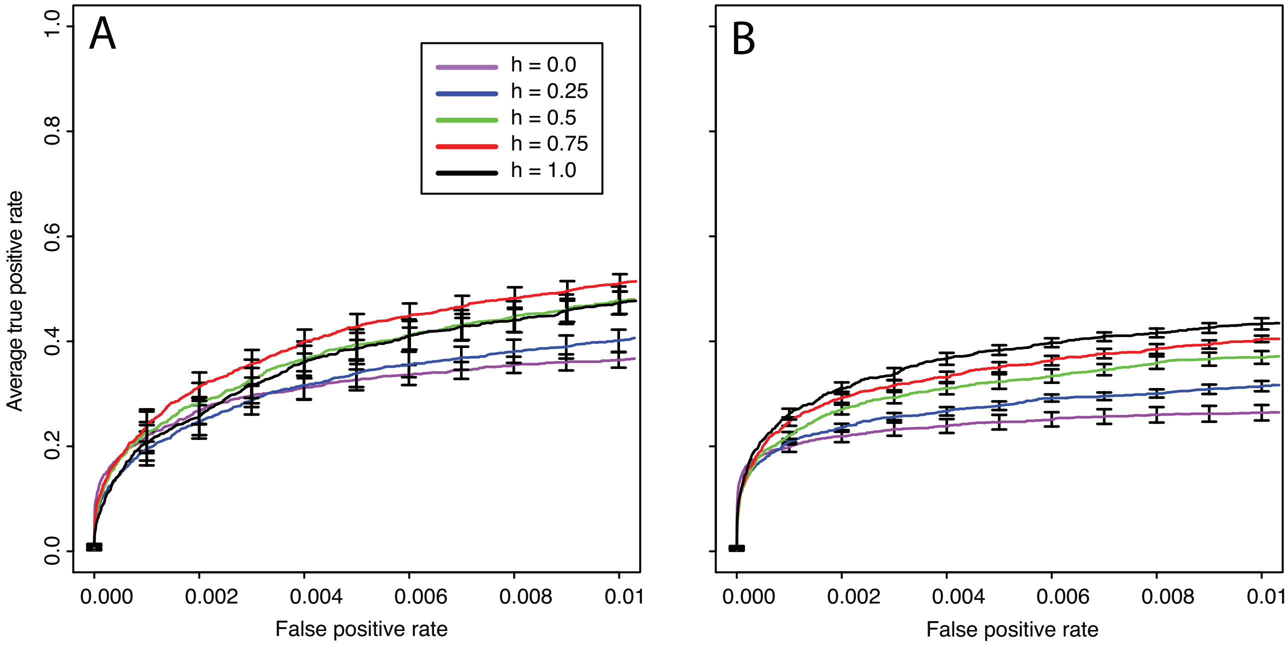

Figure 11: Influence of the dominance coefficient (h) on identification of beneficial loci with selection coefficients $s = 0.1$ (A) and $s = 0.025$ (B)

1.11 Influence of the starting allele frequency

The allele frequency change that can be expected in a single generation of selection is amongst other factors dependent on the starting allele frequency. To investigate the influence of the starting allele frequency on the power to identify beneficial alleles we used simulations with default conditions and grouped the 150 beneficial loci according to the starting frequency into 5 bins. The average number of beneficial loci in the resulting frequency classes varied from 91.0 to 12.6; The starting allele frequency has a significant influence on the power to identify beneficial loci (Kruskal-Wallis rank sum test with pAUC; $s = 0.1$: $\chi^2 = 44.67$, $p = 1.70e - 08$; $s = 0.025$: $\chi^2 = 50.87$, $p = 9.18e - 10$). Not unexpectedly, we observed the highest performance for loci starting at intermediate allele frequencies $(0.1 - 0.6)$ for both, strongly and weakly selected loci (supplementary fig. 12). Both, loci starting at low and high frequencies, are expected to yield only comparatively low allele frequency differences between the base and the evolved population because the allele frequency difference per generation is proportional to the product of the major and the minor allele frequency (pq). If neutrally evolving loci will due to chance result in similar allele frequency differences than the beneficial loci, the performance will be decreased.

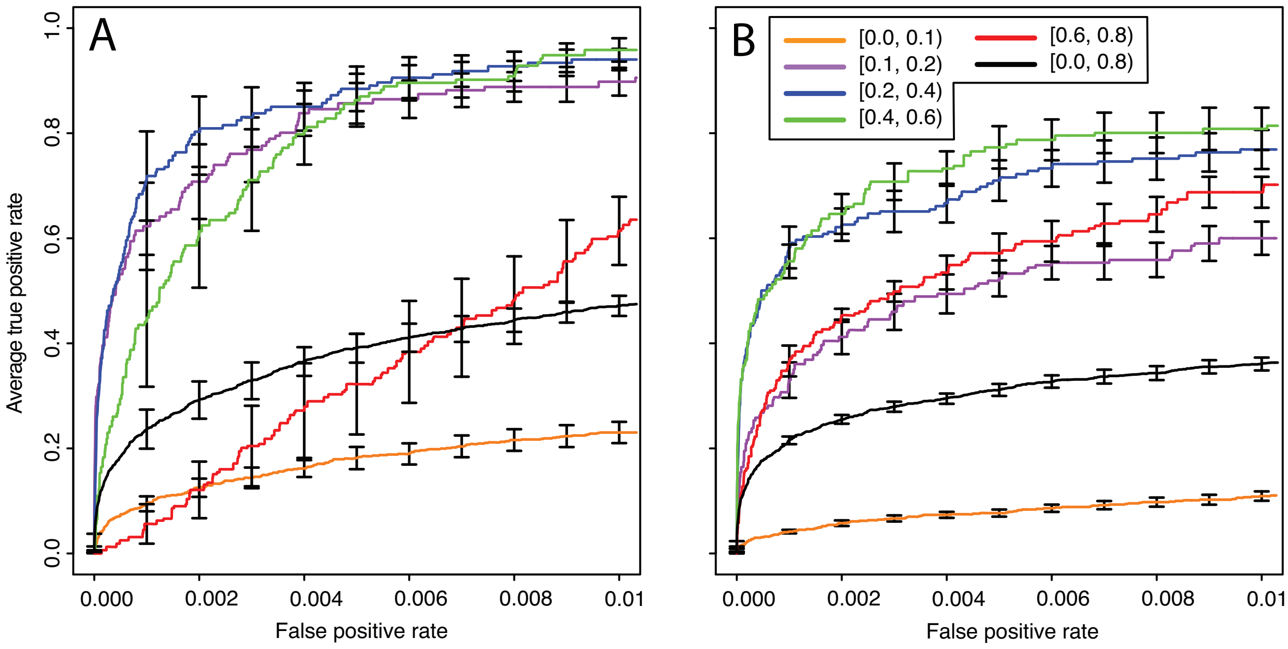

Figure 12: Influence of the starting allele frequency on identification of beneficial loci with selection coefficients $s = 0.1$ (A) and $s = 0.025$ (B)

1.12 Influence of mixing beneficial loci with different selection coefficient

We found that with a high budget study design ($N = 2000$, $generations = 120$, $replicates = 10$; see text of main manuscript) beneficial loci with a selection coefficient as low as $s = 0.005$ may be identified (main manuscript figure 7). This result is, however, only valid if all beneficial loci have identical selection coefficients. In an actual E&R study beneficial loci will most likely be composed of a mixture of loci having very different selection coefficients. We, therefore, evaluated the effect of mixing loci with different selection coefficient on the power to identify beneficial loci. We generated two sets of 150 beneficial loci with mixed selection coefficients, one including loci with $s = 0.5$ and one excluding loci with $s = 0.5$ (supplementary table 2). Loci with selection coefficients of $s = 0.001$ were not included in either set, as they could not be identified at unmixed conditions with the high budget study design. To evaluate the effect of mixing beneficial loci we compared the performance of the mixed set of loci to unmixed conditions (150 beneficial loci of identical selection coefficient as shown in the main manuscript in fig. 7).

We found that mixing loci of different selection coefficients decreases the power to identify beneficial loci relative to unmixed conditions (supplementary fig. 13). As compared to unmixed sets, the performance was increased only for loci with the highest selection coefficient in each of the mixed sets. This can be easily explained by the fact that reduced numbers of strongly selected loci result in an increased performance (supplementary fig. 10). In mixed sets of beneficial alleles only loci with $s = 0.05$ could be identified using a high budget study design as compared to loci with $s = 0.005$ in unmixed sets (supplementary fig. 13).

Table 2: Composition of the two mixed sets of beneficial loci. One set included loci with a selection coefficient (s) of $s = 0.5$ (incl.) and one set excluded loci with $s = 0.5$ (excl). Given numbers are counts of loci.

s	incl.	excl.
0.5	30	0
0.1	30	37
0.05	30	38
0.01	30	37
0.005	30	38
sum	150	150

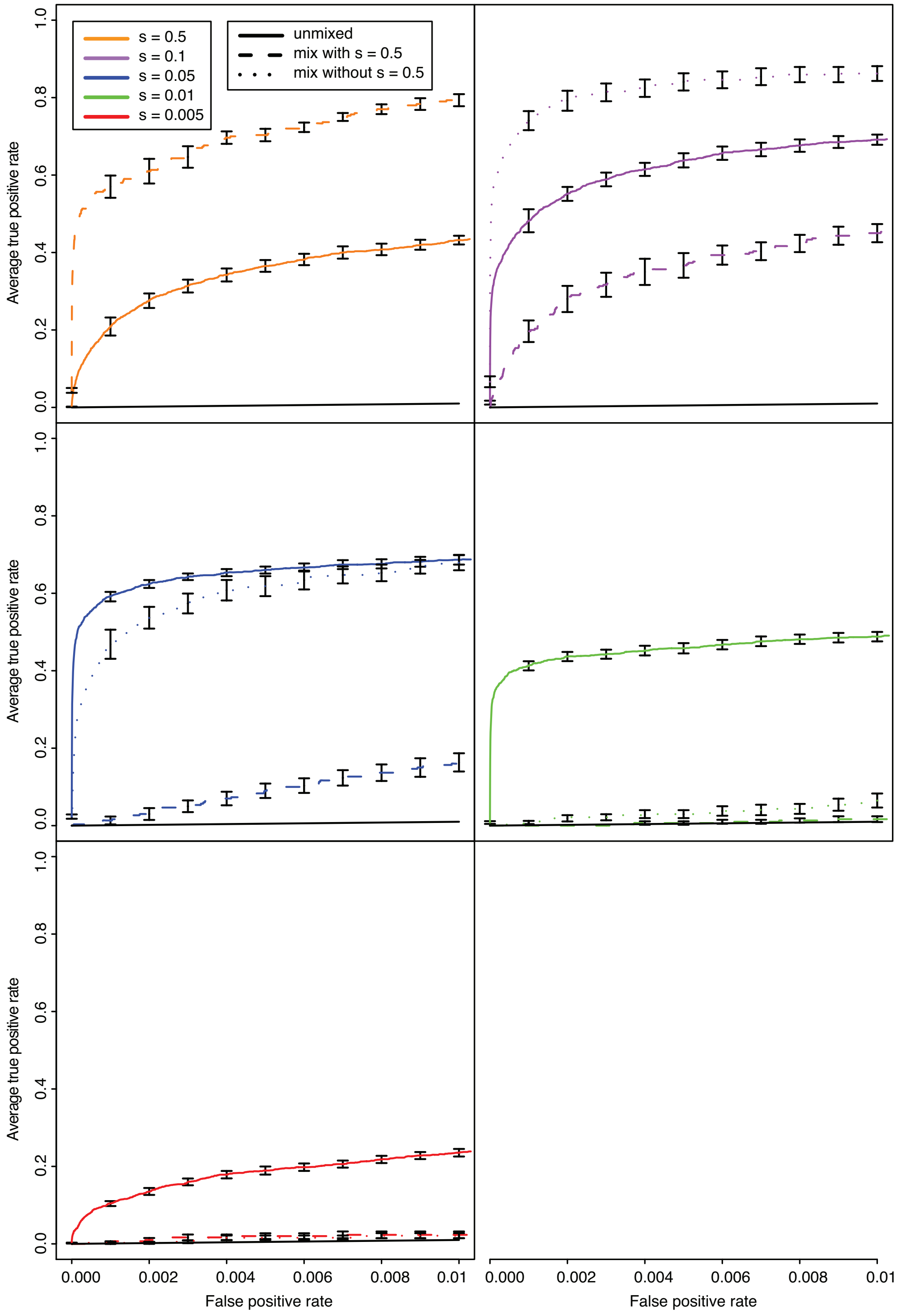

Figure 13: Influence of mixing beneficial loci with different selection coefficients on the power to identify beneficial loci. Solid lines represent unmixed sets of 150 beneficial loci, whereas the dashed lines represent the two mixed sets of beneficial loci. Black lines indicates the null hypothesis of a random classifier. See supplementary table 2 for composition of the mixed sets of beneficial loci.

1.13 Influence of estimating the allele frequency with Pool-Seq

Our results are based on the actual allele frequencies in populations, which may for example be obtained by sequencing all individuals in a population separately. Most E&R studies however have relied on Pool-Seq, which is a cost effective approach, to estimate the allele frequencies [3, 5, 6, 8]. To investigate the influence of estimating the allele frequencies with Pool-Seq we simulated the sampling properties of Pool-Seq with different average coverages ranging from 10 to 200. The major quantiles of the resulting distribution of coverages can be found in table 3.

The coverage has a significant influence on the power to identify beneficial loci when allele frequencies are estimated with Pool-Seq (Kruskal-Wallis rank sum test with pAUC; $s = 0.1$: $\chi^2 = 23.04$, $p = 0.00033$; $s = 0.025$: $\chi^2 = 54.75$, $p = 1.5e - 14$;). For the identification of strongly selected loci an average coverage of about 50 is sufficient (Fig. 14). By contrast, increasing the average coverage to about 200 improves the ability to identify weakly selected loci (Fig. 14). Based on these results, we suggest that average coverages >200 will be necessary in order to reliably detected weakly selected loci.

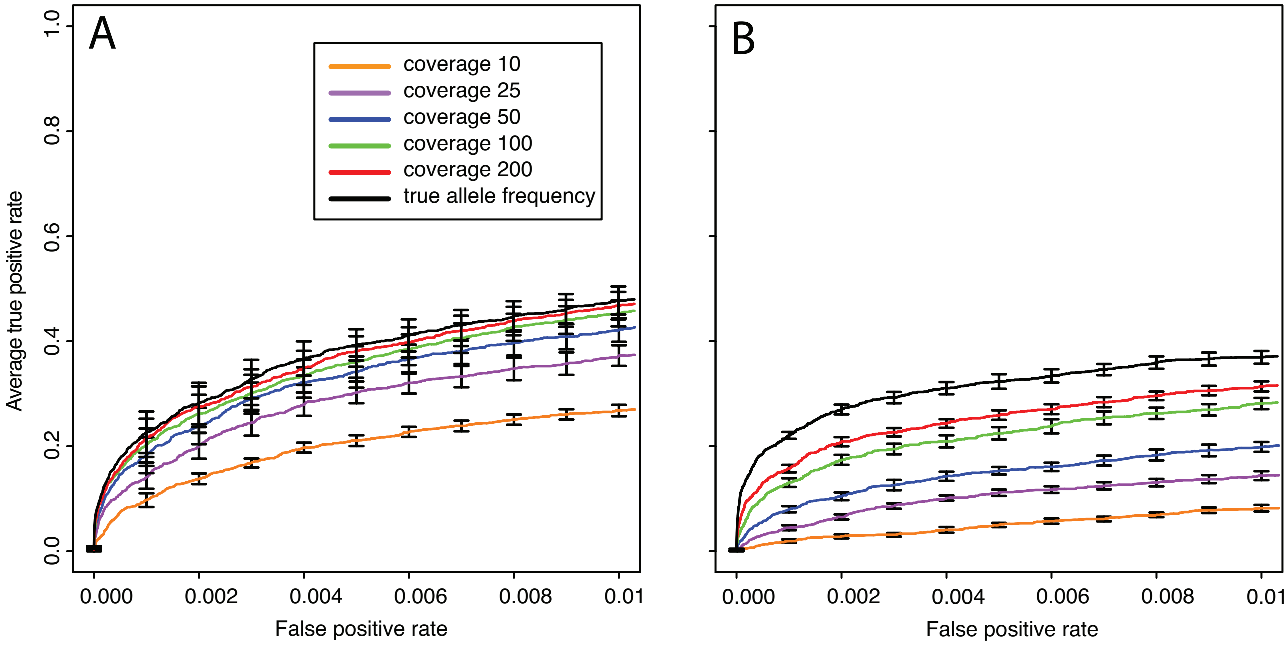

Figure 14: Influence of the coverage of Pool-Seq on the identification of beneficial loci with selection coefficients $s = 0.1$ (A) and $s = 0.025$ (B)

Table 3: Coverage distribution for different targeted average coverages ($av.cov$). Quantiles of the coverage (ranging from 2.5 to 97.5) and the mean of the coverage (μ) are shown for strongly ($s = 0.1$) and weakly selected ($s = 0.025$) loci.

		quantile [%]							μ
s	$av.cov$	2.5	5	10	50	90	95	97.5	
0.1	10	1.13	2.03	3.20	8.77	16.79	19.46	21.87	10.00
	25	9.72	11.51	13.76	23.06	35.25	39.25	42.86	24.42
	50	27.21	30.16	33.77	48.44	66.11	71.42	76.04	49.82
	100	69.40	73.91	79.29	99.87	122.57	129.21	135.03	101.00
	200	151.96	158.98	167.35	198.73	232.21	241.97	250.54	199.88
0.025	10	1.79	2.76	4.05	9.85	17.96	20.59	22.92	11.04
	25	10.06	11.98	14.32	23.98	35.79	39.42	42.63	25.13
	50	27.47	30.54	34.29	49.29	67.16	72.61	77.43	50.65
	100	66.78	71.48	77.16	99.02	122.52	129.23	135.08	100.03
	200	151.43	158.18	166.15	195.85	228.88	238.85	247.66	197.34

2 Supplementary figures

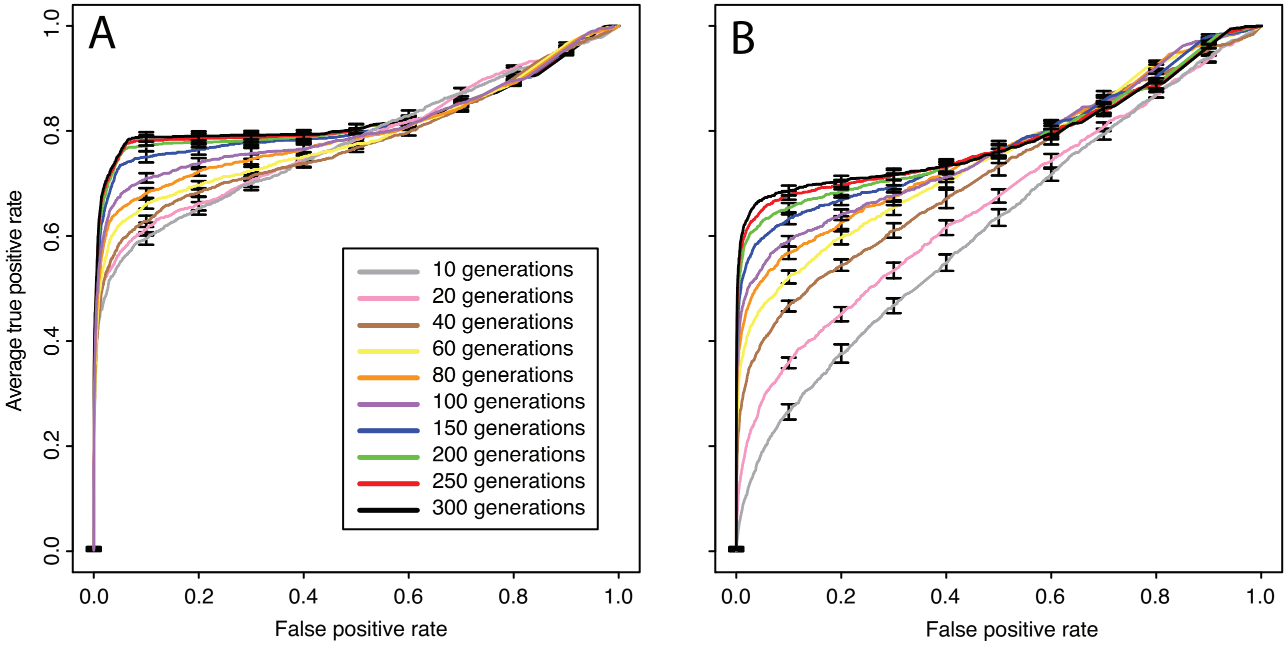

Figure 15: Influence of the number of generations on the identification of beneficial loci with selection coefficients of $s = 0.1$ (A) and $s = 0.025$ (B)

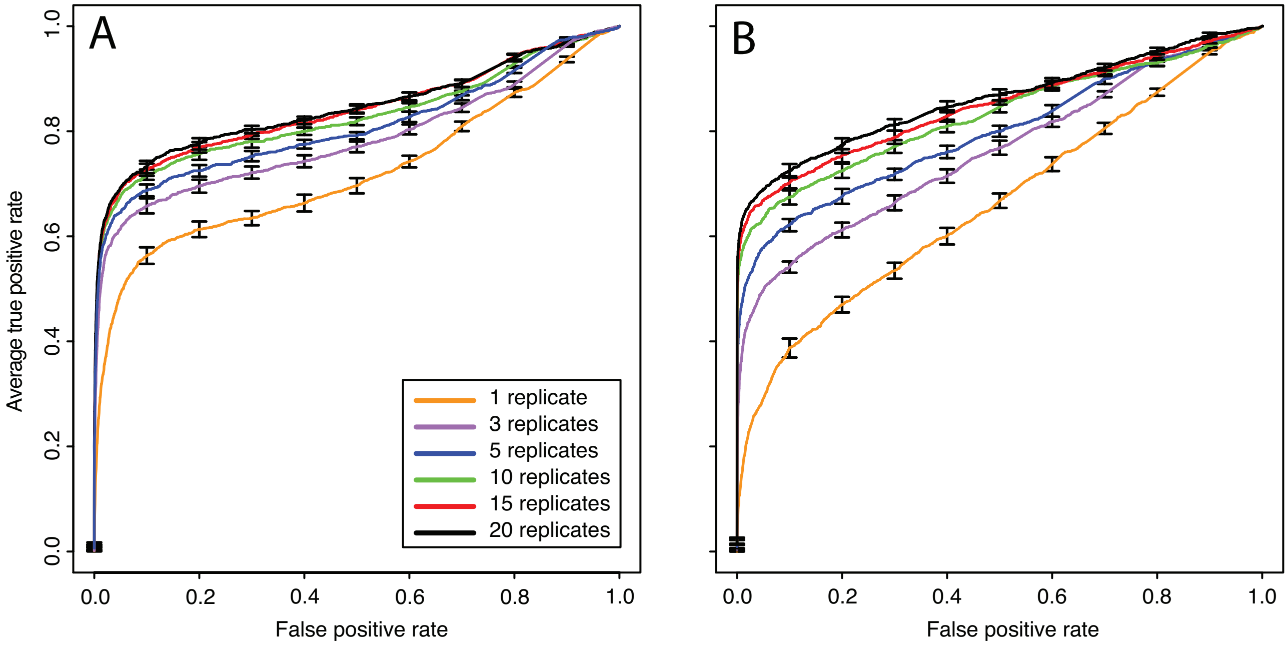

Figure 16: Influence of the number of biological replicates on the identification of beneficial loci with selection coefficients of $s = 0.1$ (A) and $s = 0.025$ (B)

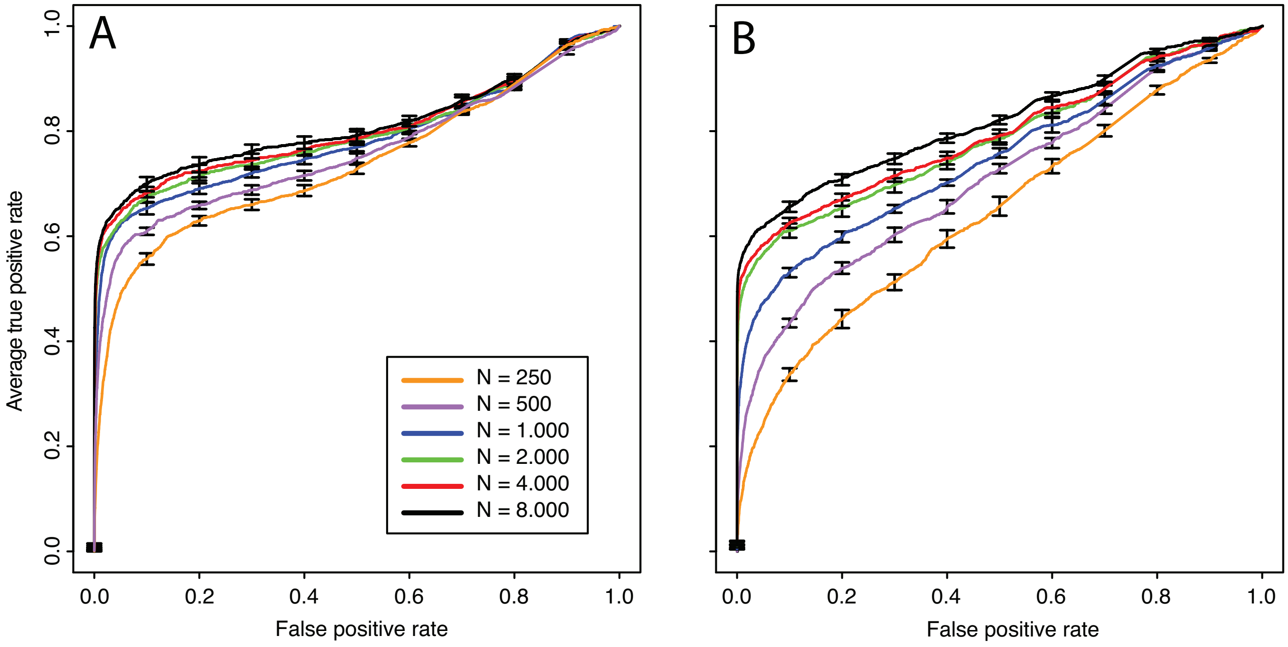

Figure 17: Influence of the population size on the identification of beneficial loci with selection coefficients of $s = 0.1$ (A) and $s = 0.025$ (B)

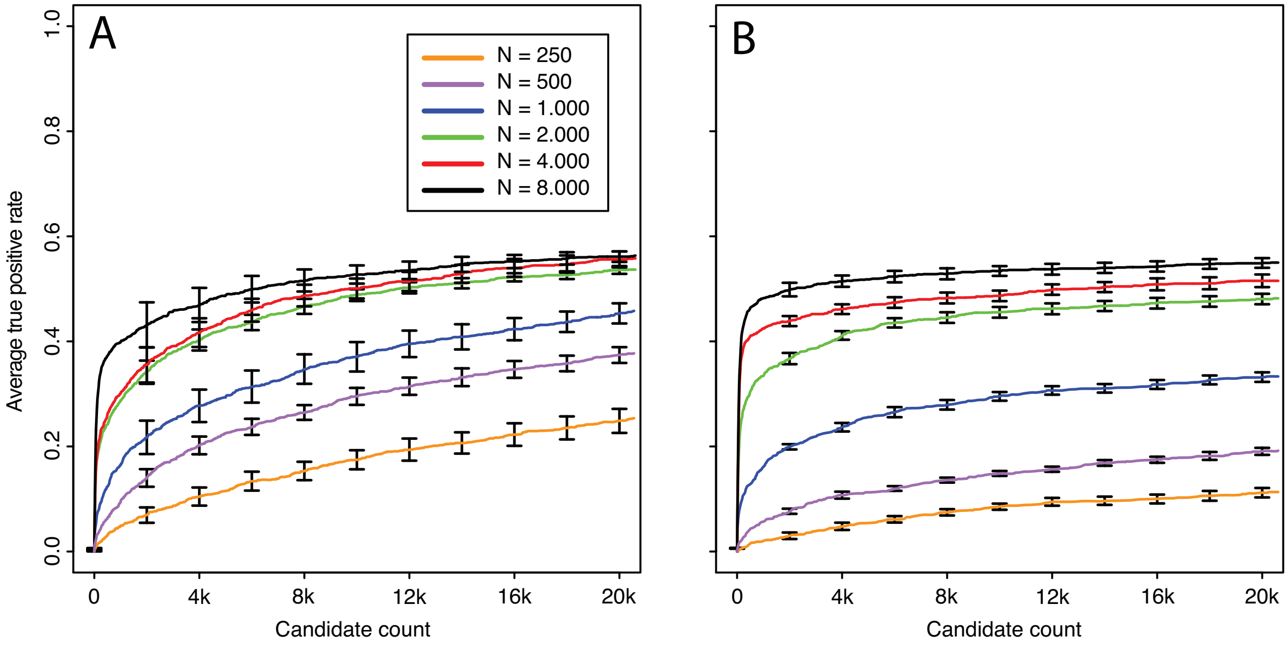

Figure 18: Influence of the population size on the identification of beneficial loci with selection coefficients of $s = 0.1$ (A) and $s = 0.025$ (B) for the 20,000 most significant loci.

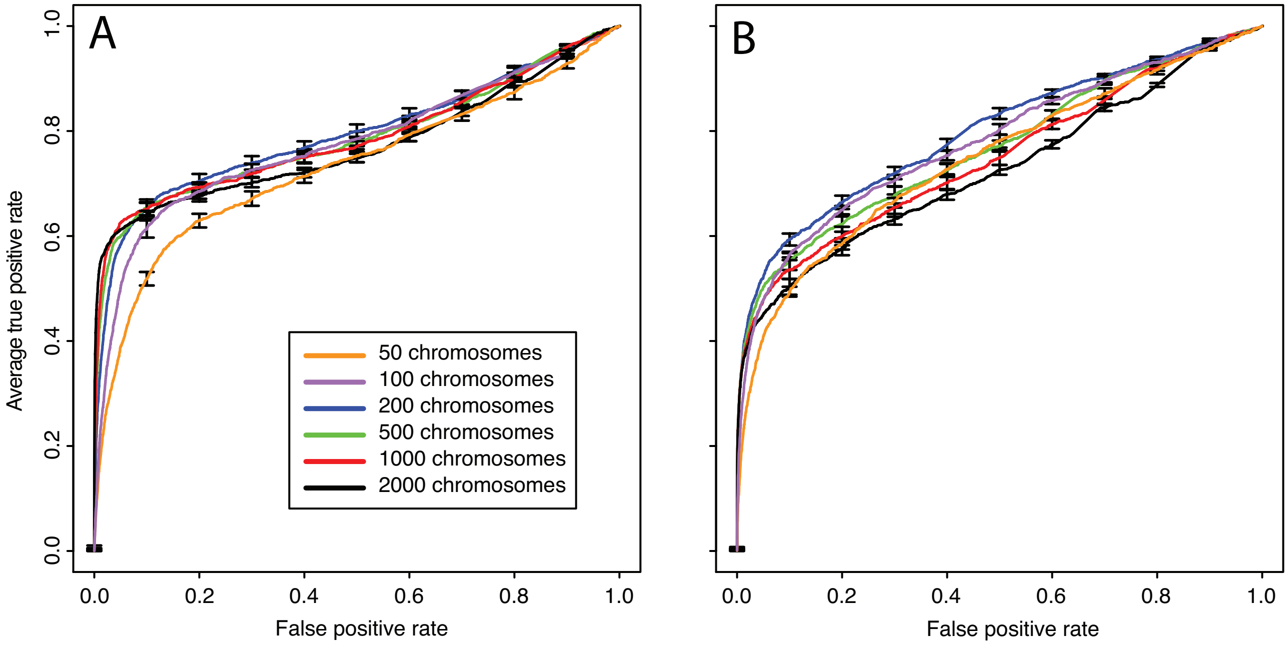

Figure 19: Influence of the number of haploid genomes in the base population on the identification of beneficial loci with selection coefficients of $s = 0.1$ (A) and $s = 0.025$ (B)

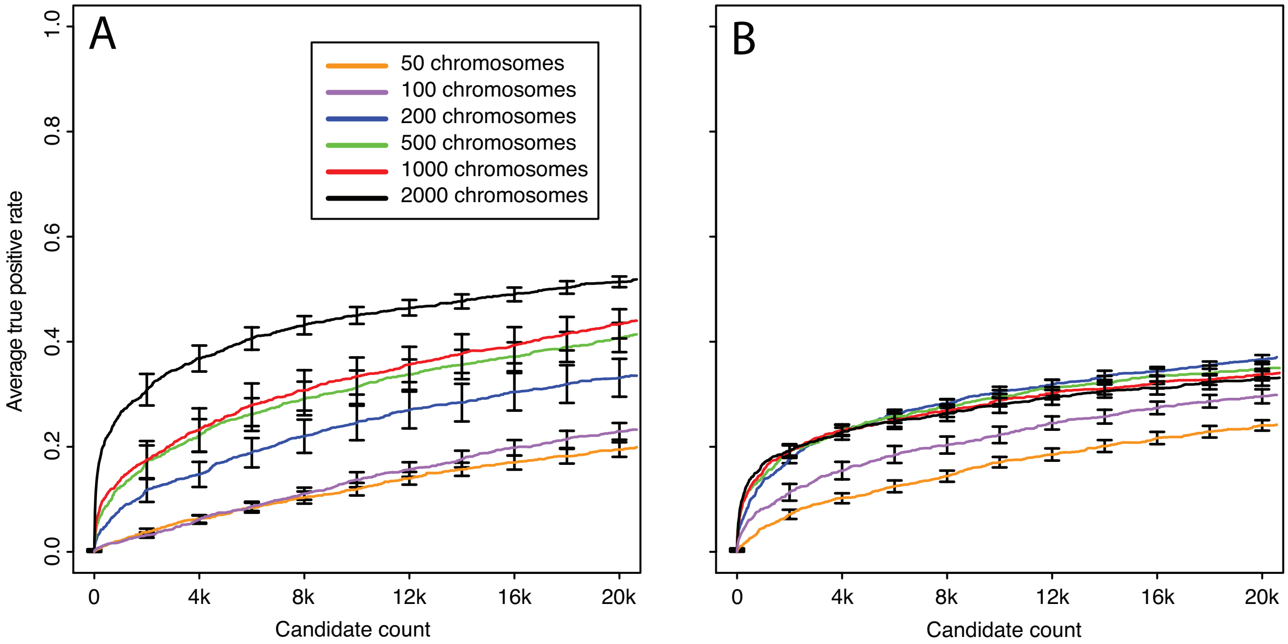

Figure 20: Influence of the number of haploid genomes in the base population on the identification of beneficial loci with selection coefficients of $s = 0.1$ (A) and $s = 0.025$ (B) for the 20,000 most significant loci.

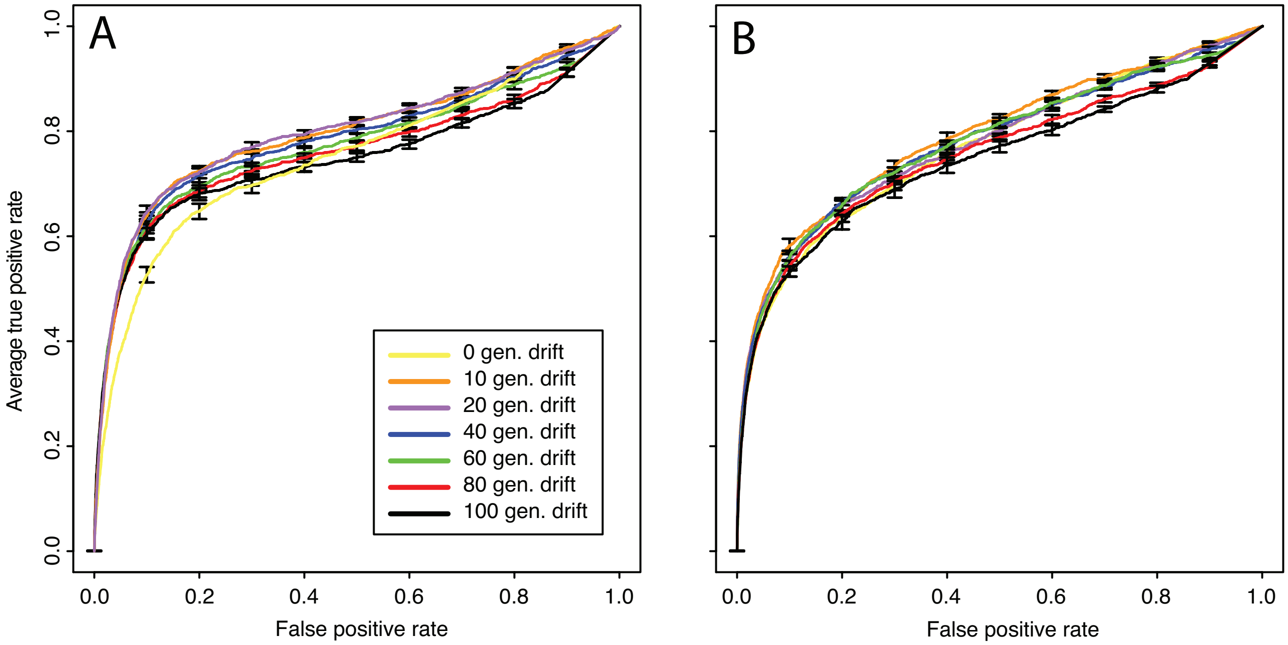

Figure 21: Influence of drift prior to experimental evolution on the identification of beneficial loci with selection coefficients of $s = 0.1$ (A) and $s = 0.025$ (B)

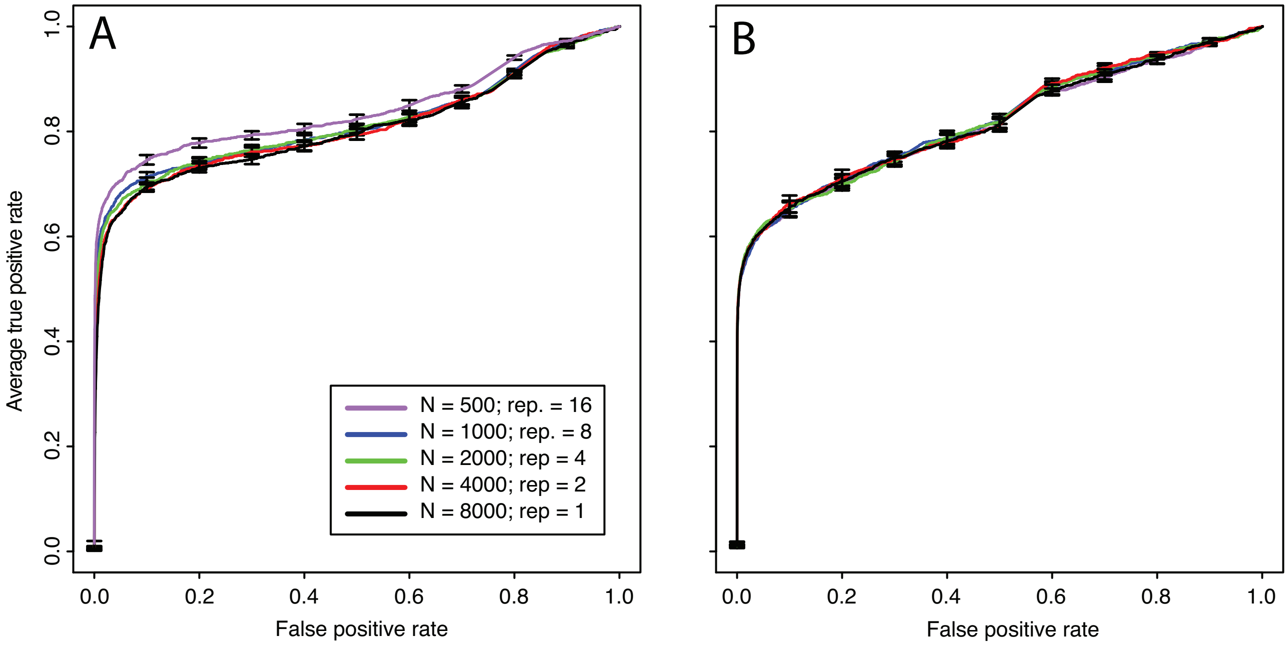

Figure 22: Tradeoff between population size and number of replicates for beneficial loci with the selection coefficients of $s = 0.1$ (A) and $s = 0.025$ (B)

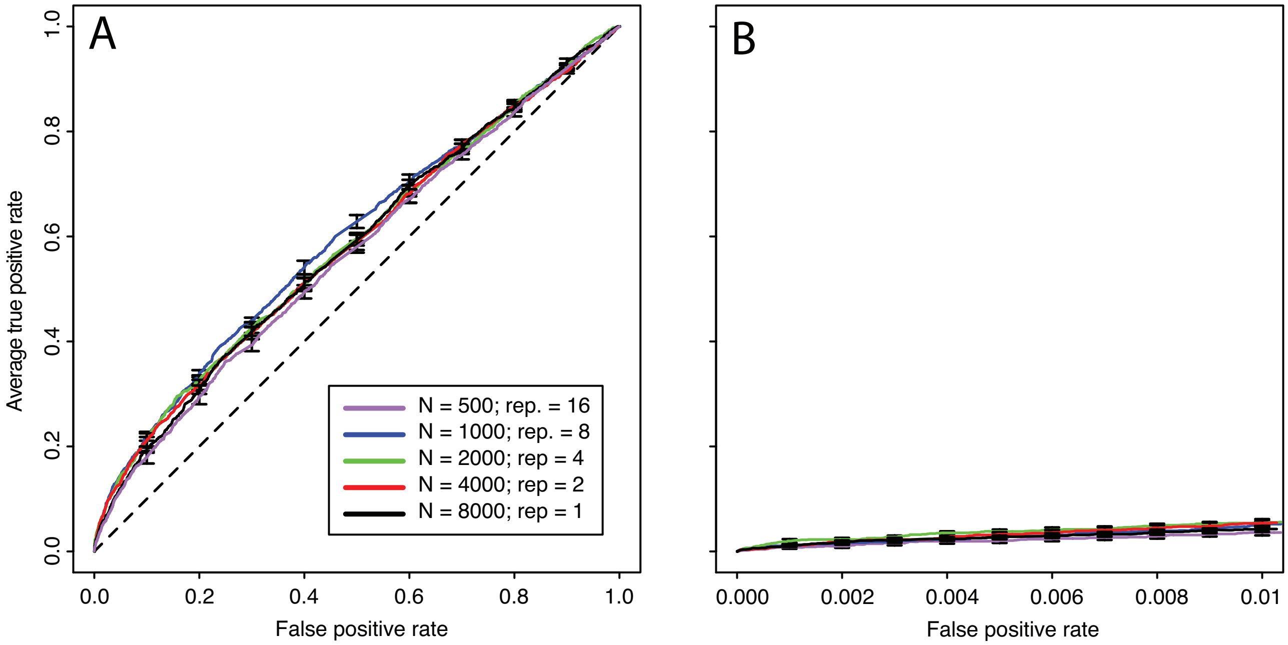

Figure 23: Tradeoff between population size and number of replicates for beneficial loci having a selection coefficient of $s = 0.0005$ with a $FPR \leq 1$ (A) and $FPR \leq 0.01$ (B); The dashed line indicates the null expectations for the power to identify beneficial loci.

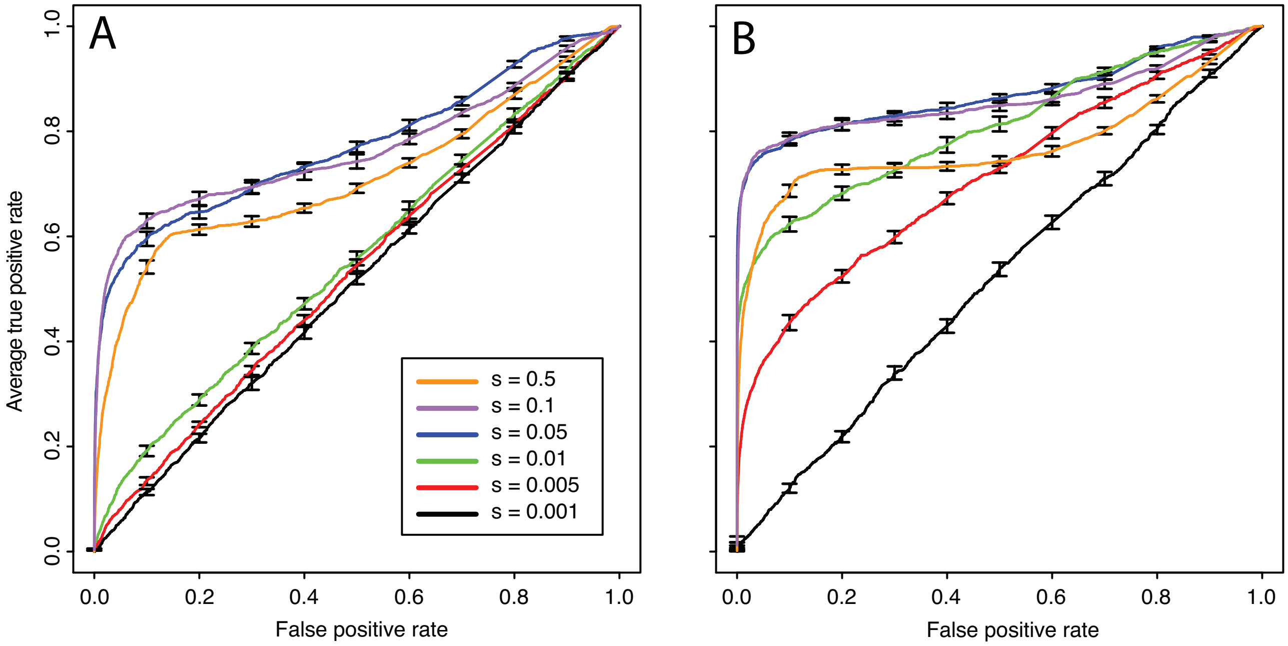

Figure 24: Power to identify beneficial loci of different effect sizes with a low budget (A) and a high budget (B) study. See text of main manuscript for explanations of low budget and high budget study.

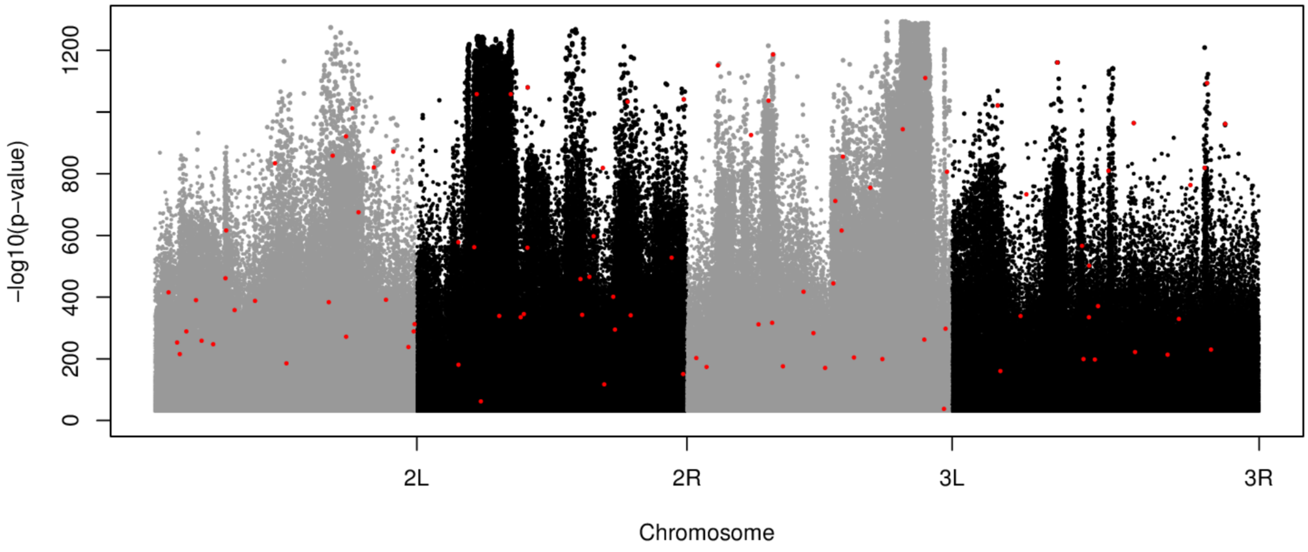

Figure 25: Example of a Manhattan plot from the low budget study design with 150 beneficial SNPs (red) having a selection coefficient of $s = 0.5$

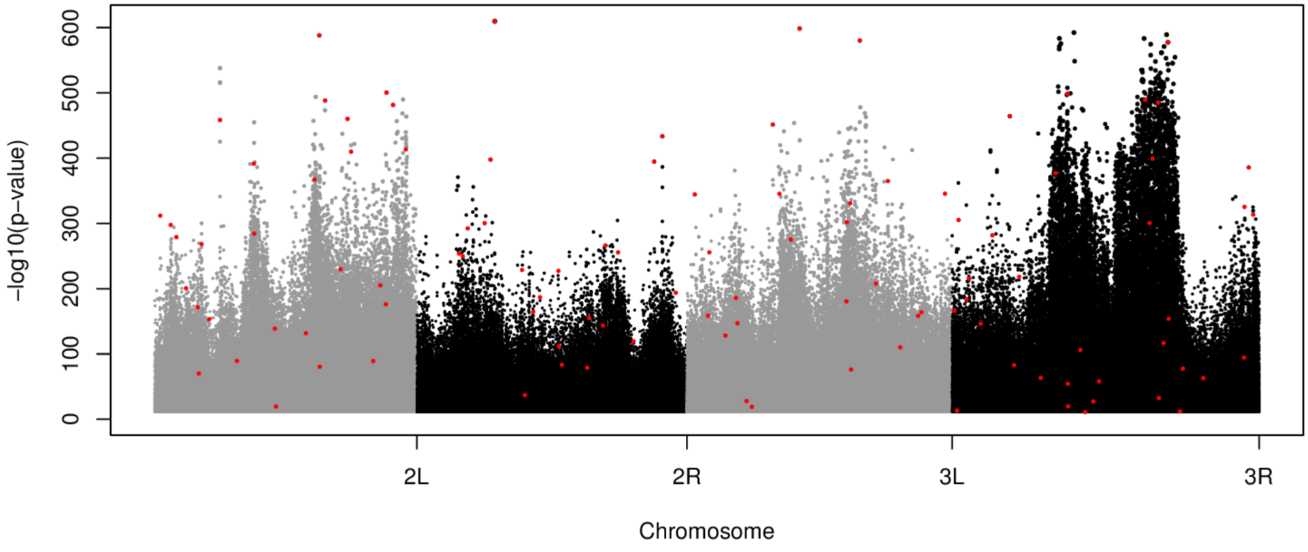

Figure 26: Example of a Manhattan plot from the low budget study design with 150 beneficial SNPs (red) having a selection coefficient of $s = 0.1$

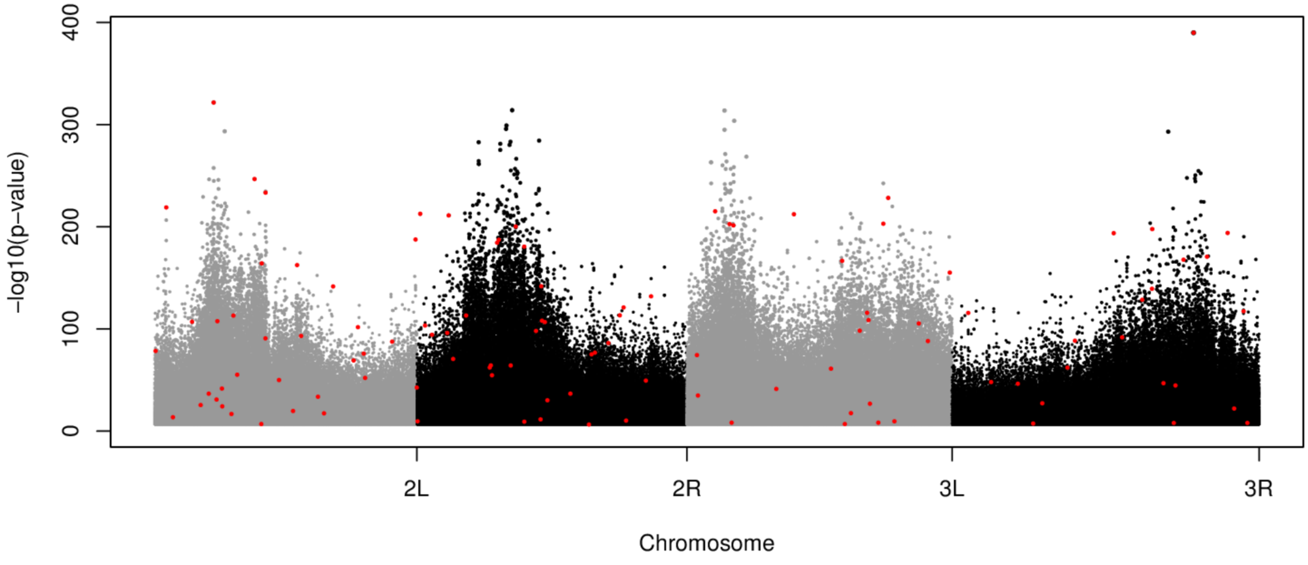

Figure 27: Example of a Manhattan plot from the low budget study design with 150 beneficial SNPs (red) having a selection coefficient of $s = 0.05$

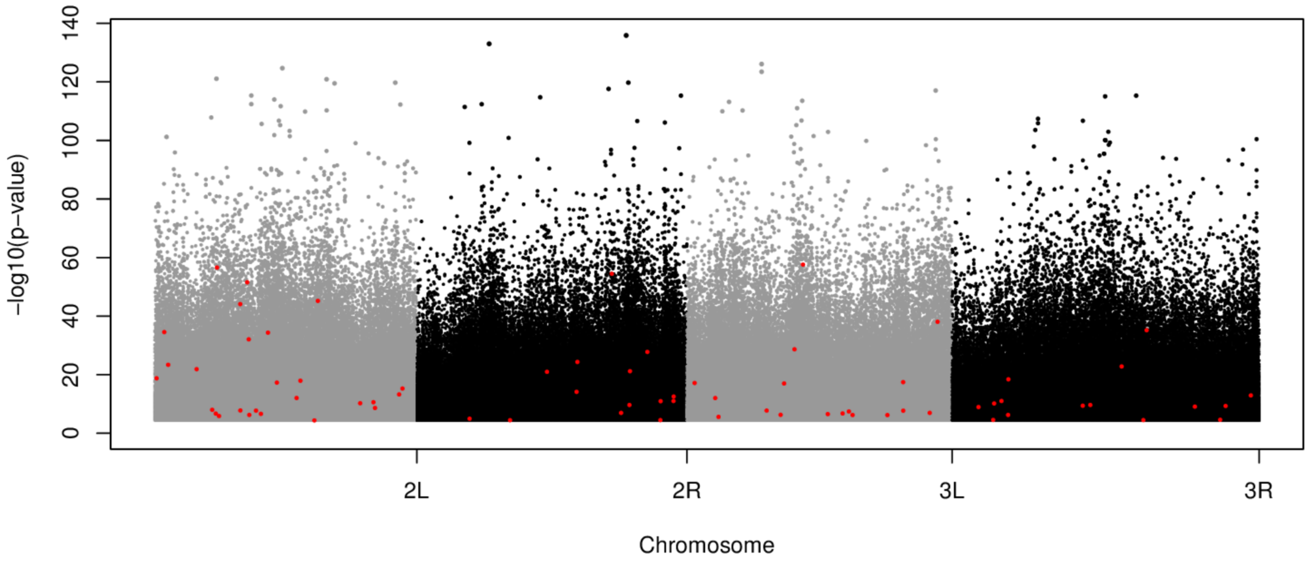

Figure 28: Example of a Manhattan plot from the low budget study design with 150 beneficial SNPs (red) having a selection coefficient of $s = 0.01$

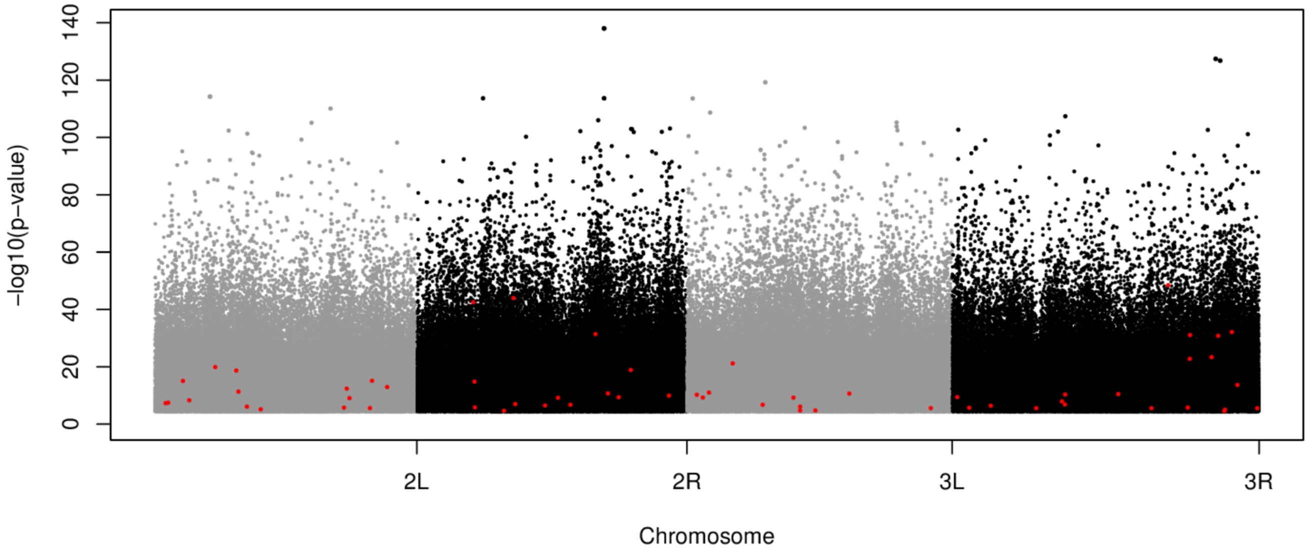

Figure 29: Example of a Manhattan plot from the low budget study design with 150 beneficial SNPs (red) having a selection coefficient of $s = 0.005$

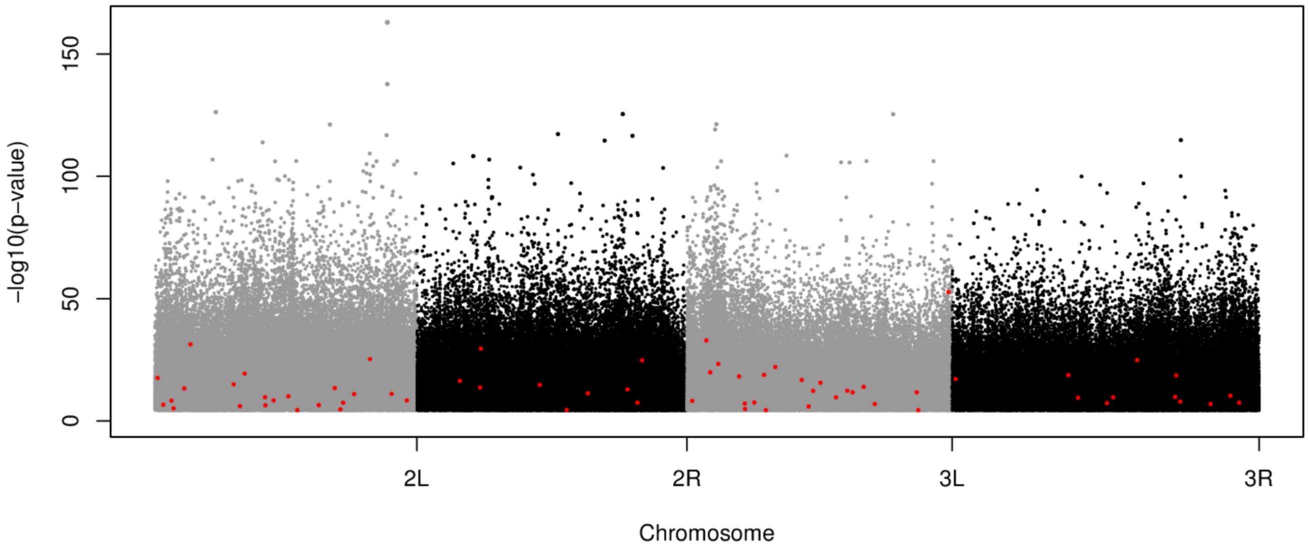

Figure 30: Example of a Manhattan plot from the low budget study design with 150 beneficial SNPs (red) having a selection coefficient of $s = 0.001$

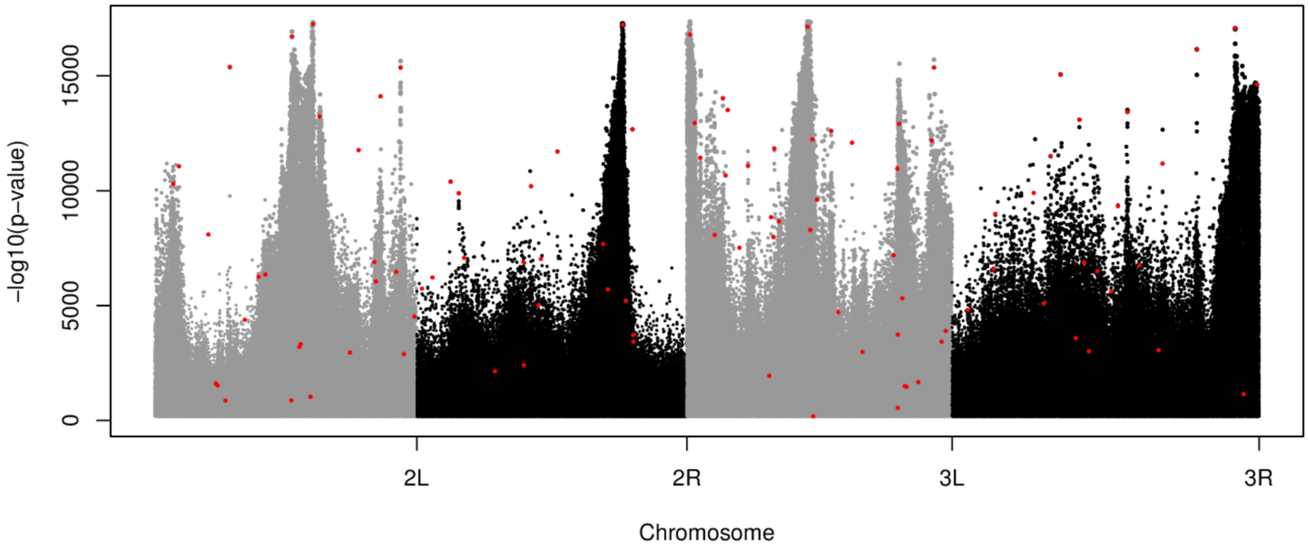

Figure 31: Example of a Manhattan plot from the high budget study design with 150 beneficial SNPs (red) having a selection coefficient of $s = 0.5$

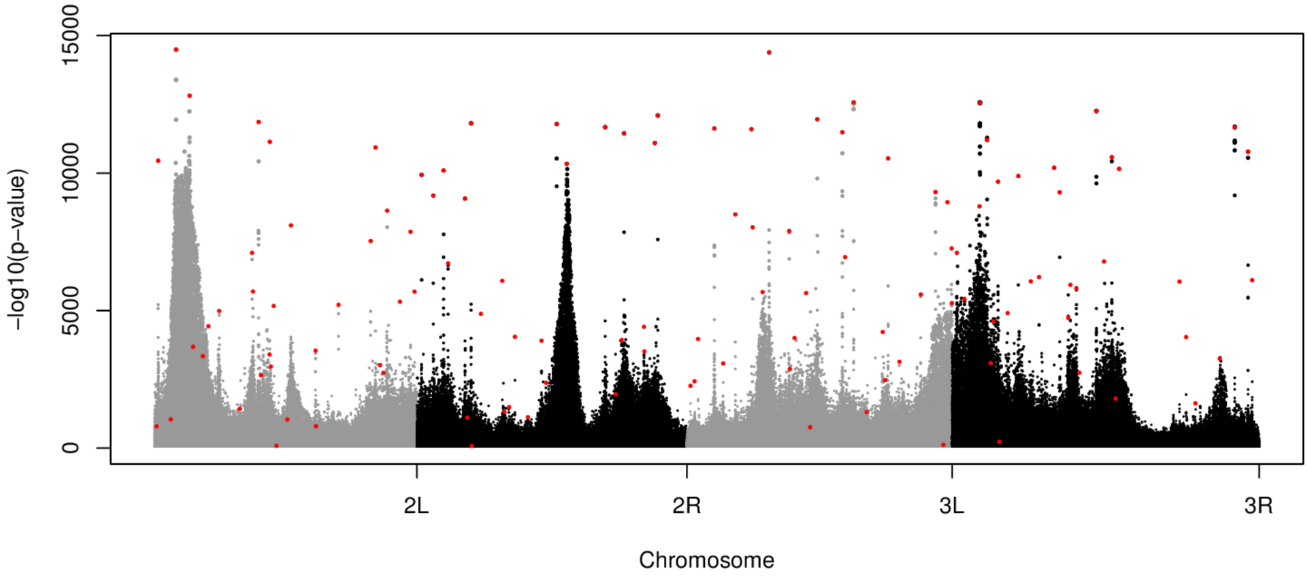

Figure 32: Example of a Manhattan plot from the high budget study design with 150 beneficial SNPs (red) having a selection coefficient of $s = 0.1$

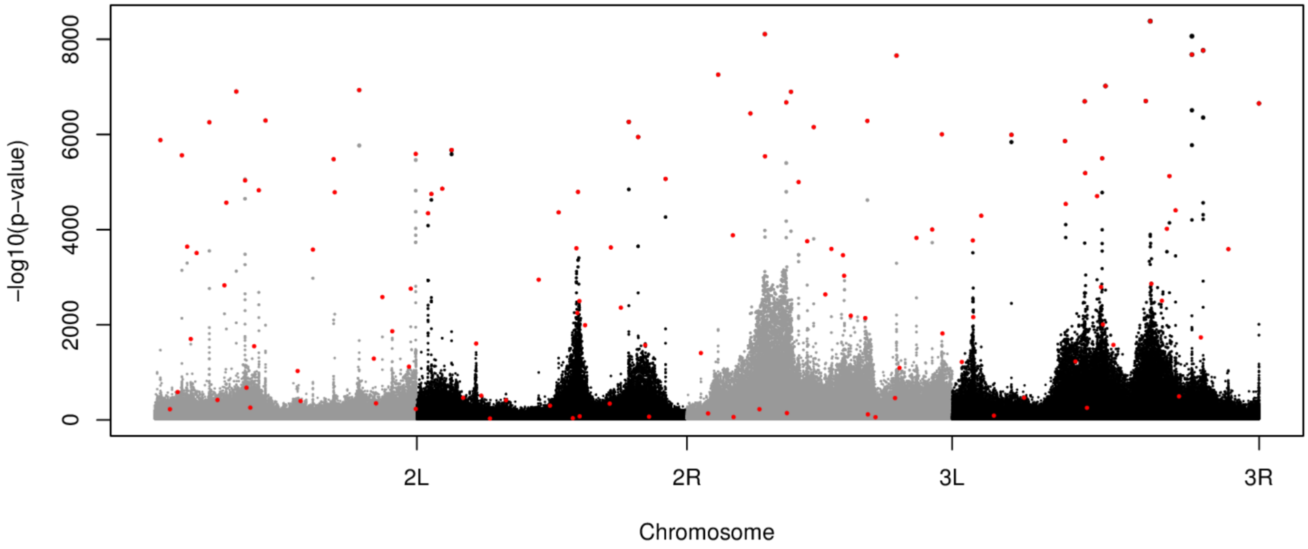

Figure 33: Example of a Manhattan plot from the high budget study design with 150 beneficial SNPs (red) having a selection coefficient of $s = 0.05$

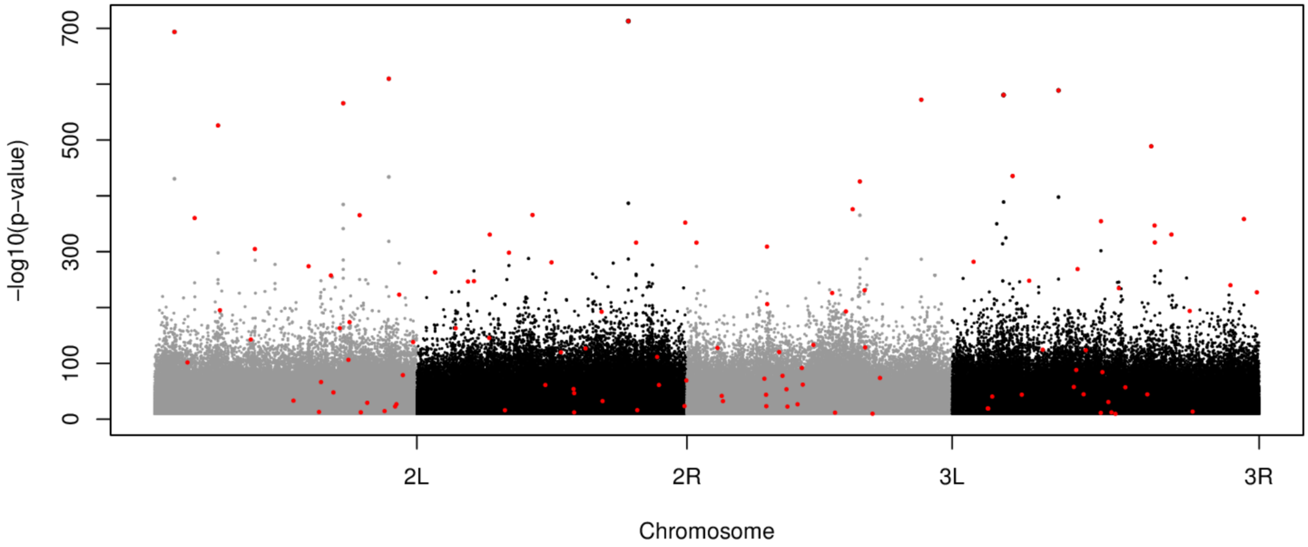

Figure 34: Example of a Manhattan plot from the high budget study design with 150 beneficial SNPs (red) having a selection coefficient of $s = 0.01$

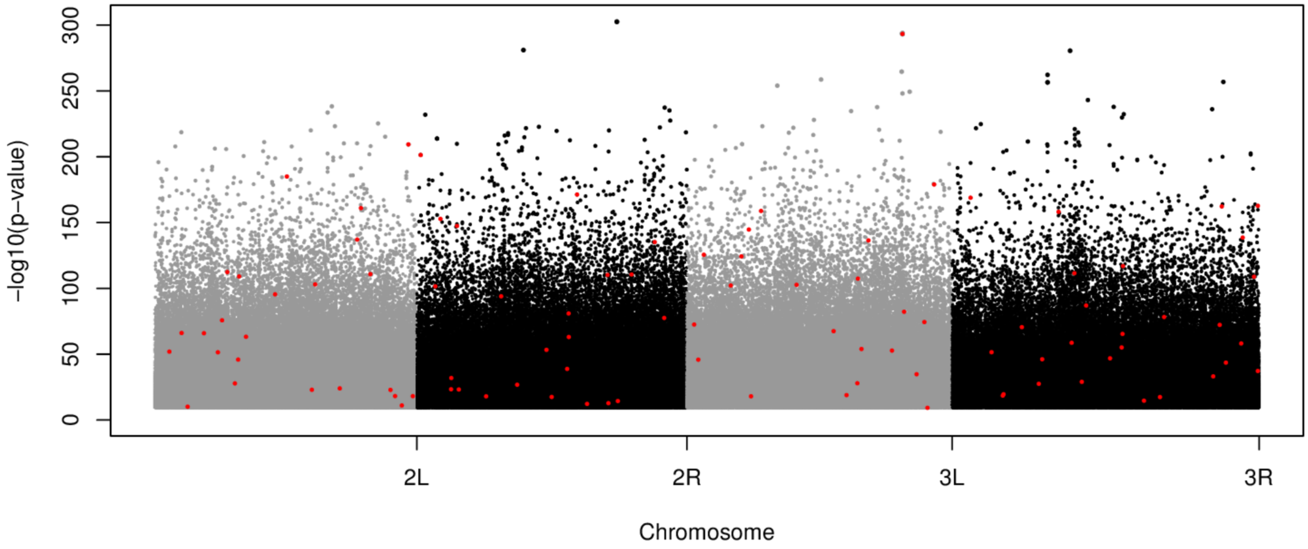

Figure 35: Example of a Manhattan plot from the high budget study design with 150 beneficial SNPs (red) having a selection coefficient of $s = 0.005$

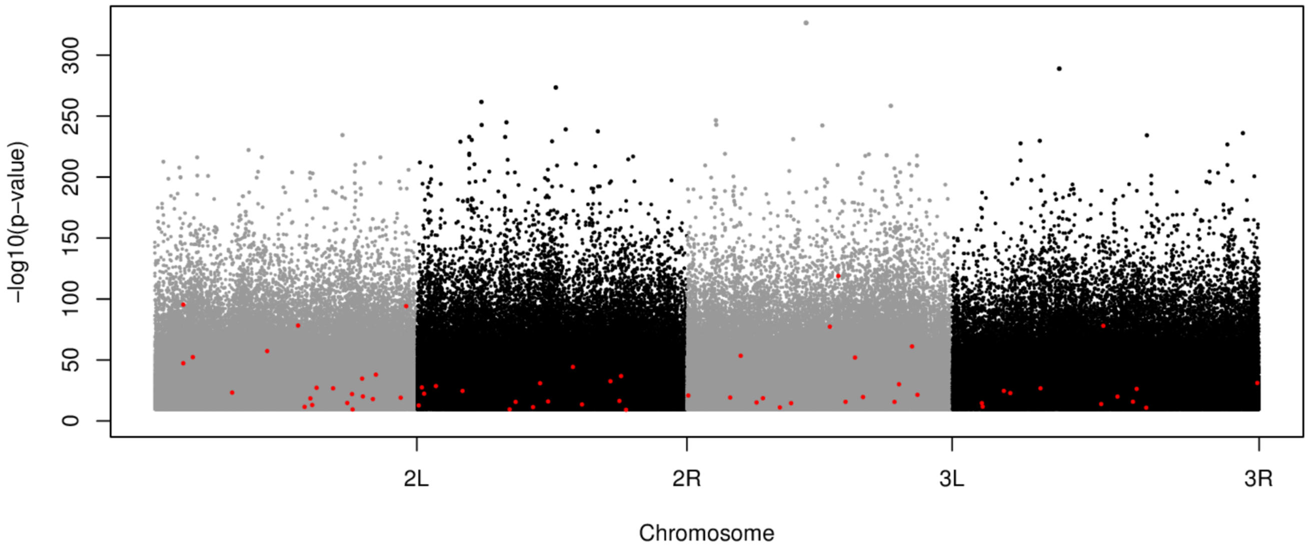

Figure 36: Example of a Manhattan plot from the high budget study design with 150 beneficial SNPs (red) having a selection coefficient of $s = 0.001$

3 Supplementary Material and Methods

3.1 Estimating the efficacy of selection

To estimate the efficacy of selection with different numbers of selected loci we used 'MimicrEELimits'. First beneficial loci with the given parameters (selection coefficient and dominance coefficient) are randomly picked from the base population where the minor allele frequency of selected loci was not allowed to exceed 0.8. Subsequently forward simulations, as described in the main text, will be performed until all loci are either fixed or lost. Given the total number of beneficial loci (c_a) and the number of fixed loci in the forward simulations (c_s) the probability of fixation (π) can be calculated as: $\pi = c_s/c_a$. For every set of beneficial SNPs, MimicrEELimits also performs neutral forward simulations (by setting the selection coefficient to zero) in order to estimate the null hypothesis. Given the total number of beneficial loci (c_a) and the number of loci fixed in the neutral simulations (c_n) the probability of fixation under neutrality (π_n) can be calculated as $\pi_n = c_n/c_a$.

MimimcrEELimits also records the distribution of offspring in every generation which allows to estimate the variance effective population size (N_{ev}) [7]. We are only showing N_{ev} for the first generation because here the reduction in the effective population size is the most pronounced. In summary we simulated 10, 25, 50, 75, 100, 150, 200, 500, 1,000, and 2,000 beneficial loci with strong ($s = 0.1$) and weak ($s = 0.025$) selection coefficients. Every simulation was repeated 20 times.

3.2 Simulating the sampling properties of Pool-Seq

To investigate the influence of estimating the allele frequencies with Pool-Seq on the power to identify beneficial loci we simulated the sampling properties of Pool-Seq for different targeted average coverages. We aimed to capture four properties of Pool-Seq. First, the coverage may differ among samples (a phenomenon frequently encountered with barcoding). We modeled this by choosing the average coverage for each sample from a Poisson distribution, where the mean corresponds to targeted average coverage over all samples. Second, Illumina sequencing has a GC-bias [9], where regions with elevated GC content have increased coverages. This results in a correlated coverage across samples. We modeled this by modulating the coverage at a given genomic positions in all samples by a fraction chosen from a Poisson distribution. This step was independently repeated for each genomic position. Third, to

model stochastic coverage heterogeneity, we randomly selected a number from a Poisson distribution where the mean was the product of the previous two sampling steps. This step was independently repeated for each genomic position in each sample. This third step yielded the final coverage for each genomic position in each sample. Finally, to model the uneven sampling of individuals in pools, we randomly picked alleles from the true allele frequency until the targeted coverage was obtained (binomial sampling).

References

1. Excoffier L, Foll M: **fastsimcoal: a continuous-time coalescent simulator of genomic diversity under arbitrarily complex evolutionary scenarios.** *Bioinformatics* 2011, **27**(9):1332–4.

2. Bastide H, Betancourt A, Nolte V, Tobler R, Stöbe P, Futschik A, Schlötterer C: **A Genome-Wide, Fine-Scale Map of Natural Pigmentation Variation in Drosophila melanogaster**. *PLoS Genetics* 2013, **9**(6):e1003534.

3. Orozco-terWengel P, Kapun M, Nolte V, Kofler R, Flatt T, Schlötterer C: **Adaptation of Drosophila to a novel laboratory environment reveals temporally heterogeneous trajectories of selected alleles.** *Molecular ecology* 2012.

4. Turner TL, Stewart AD, Fields AT, Rice WR, Tarone AM: **Population-based resequencing of experimentally evolved populations reveals the genetic basis of body size variation in Drosophila melanogaster.** *PLoS genetics* 2011, **7**(3):e1001336.

5. Turner TL, Miller PM: **Investigating natural variation in Drosophila courtship song by the evolve and resequence approach.** *Genetics* 2012, **191**(2):633–42.

6. Remolina SC, Chang PL, Leips J, Nuzhdin SV, Hughes KA: **Genomic basis of aging and life-history evolution in Drosophila melanogaster.** *Evolution* 2012, **66**(11):3390–403.

7. Crow JF, Denniston C: **Inbreeding and Variance Effective Population Numbers**. *Evolution***42**(3):pp. 482–495.

8. Parts L, Cubillos FA, Warringer J, Jain K, Salinas F, Bumpstead SJ, Molin M, Zia A, Simpson JT, Quail MA, Moses A, Louis EJ, Durbin R, Liti G: **Revealing the genetic structure of a trait by sequencing a population under selection.** *Genome research* 2011, **21**(7):1131–8.

9. Dohm JC, Lottaz C, Borodina T, Himmelbauer H: **Substantial biases in ultra-short read data sets from high-throughput DNA sequencing.** *Nucleic acids research* 2008, **36**(16):e105.
